# Four New Species of *Macquartia* (Diptera: Oestroidea) from China and Phylogenetic Implications of Tachinidae †

**DOI:** 10.3390/insects13121096

**Published:** 2022-11-28

**Authors:** Henan Li, Baihui Zhang, Wenya Pei, Haoran Sun, Jinliang Chen, Xinzhang Gao, Honglin Peng, Dong Zhang, Chuntian Zhang

**Affiliations:** 1 School of Ecology and Nature Conservation, Beijing Forestry University, Beijing 100083, China; 2 College of Life Science, Shenyang Normal University, Shenyang 110034, China; 3 Dalaoling Nature Reserve Administration of Yichang Three Gorges, Yichang 443000, China

**Keywords:** Calyptratae, Macquartiini, mitochondrial genome, molecular phylogeny, morphology, new species

## Abstract

**Simple Summary:**

The subfamily Tachininae comprises over 2700 species on Earth, making it the second largest group of Tachinidae, some of which have the potential to be used in the biocontrol of agricultural and forest pests. This subfamily shows the largest morphological variance, which is reflected by their great diversity in terms of ecology and host preferences. *Macquartia* belongs to Diptera, Tachinidae, and Tachininae, among which there are 12 recorded species. Whilst previous research on *Macquartia* has focused on the description of species morphology, a few studies have explored the phylogenetic relationships, but with a restricted number of species and several DNA fragments, which are insufficient to study the phylogeny of a genus, especially in order to understand the evolution of ecological traits in Tachinidae. To provide further insight into the relationships of *Macquartia*, the complete mitochondrial genomes of *M. brunneisquama* sp. nov., *M. chinensis* sp. nov., *M. flavifemorata* sp. nov., and *M. flavipedicel* sp. nov. were sequenced and compared in this study. The mitogenomes of these four *Macquartia* species were discovered to be highly conserved. Phylogenetic analyses, based on the mitogenome data of 19 species of Tachinidae and outgroups, supported the monophyly of *Macquartia* and generated basic data for the phylogenetic study of the Tachinidae.

**Abstract:**

*Macquartia* Robineau-Desvoidy (Diptera: Tachinidae, Tachininae) represents one of the most ancient evolutionary lineages of tachinids, parasitizing Chrysomelidae larvae. We found four new *Macquartia* species collected by malaise traps, namely *M. brunneisquama* sp. nov., *M. chinensis* sp. nov., *M. flavifemorata* sp. nov., and *M. flavipedicel* sp. nov. These new species are described and illustrated, and their comparison with congeners as well as an identification key to the 12 species of *Macquartia* from China known to date are included. To determine the significance of the mitogenome architecture and evolution across different tachinid lineages of this primitive taxonomic group, four complete mitochondrial genomes were sequenced, annotated, and analyzed. The gene arrangements are consistent with the ancestral insect mitogenomes. The full-length sequences and protein-coding genes (PCGs) of the mitogenomes of the four species are all AT-biased. Analyses of Ka/Ks and overall *p*-genetic distance demonstrated that *nad5* showed the highest evolutionary rate and *nad1*/*nad4L* were the most conserved genes among the four species. Phylogenetic reconstruction based on 13 PCGs strongly supported the monophyly of *Macquartia*, and the relationships of the four species are (*M. flavifemorata* + (*M. flavipedicel* + (*M. brunneisquama + M. chinensis*))). This study will help enhance our understanding of the taxonomic status and phylogenetic relationships in Tachinidae.

## 1. Introduction

Tachinidae are one of the most morphologically diverse yet evolutionarily recent families of Diptera with 1248 described species in China [1]. They are the largest parasitoid group of insects besides parasitic wasps, including both ecologically and economically important species. Tachininae are the most morphologically diverse subfamily of tachinids, including a number of disparate tribes. *Macquartia* Robineau-Desvoidy (Diptera: Tachinidae) is a genus from the tribe Macquartiini, with 25 species that are widely distributed in the Afrotropical, Oriental, Palearctic and Nearctic regions, and counting eight species recorded in China [1,2,3].

Species of *Macquartia* with a known biology are parasitoids of Coleoptera larvae dwelling in Chrysomelidae [4]. Cerretti proposed that the ancestral tachinid was probably a parasitoid of beetles based on a morphological phylogeny [5]. Stireman reported that the tribes Macquartiini and Myiophasiini are sister groups to all other Tachinidae, as almost all early clades of Tachinidae are possibly oviparous, from which ovolarviparous lineages arose several times separately [6]. Under this reconstruction, shifts towards ovolarvipary would have occurred independently in the following branches: Macquartiini + Myiophasiini, Palpostomatini + Imitomyia, Dexiinae, Strongygastrini, Tachininae, and at least six times within the Exoristinae. These studies generally support the hypothesis that the ancestral host of tachinids was probably a beetle, and transitions to Lepidoptera and caterpillars as hosts may have spurred tachinid diversification. *Macquartia* has a close phylogenetic relationship with *Porphyromus* and Myiophasiini (*Gnadochaeta*, *Cholomyia*) based on molecular evidence. Herting and Dely-Draskovits synonymized many genera from the Palearctic Region under *Macquartia*. Different systematic treatments of *Macquartia* are mainly due to morphological similarity among the genera of *Macquartia* and *Dufouria*, *Melastrongygaster, Gnadochaeta*, *Porphyromus*, and Myiophasiini, as well as to a lack of molecular evidence in 1993 [1,7]. We follow the treatment of *Macquartia* and characterize the genus with a new generic definition [8,9,10]. The higher-level phylogeny of longhorn beetles (Coleoptera: Chrysomeloidea) was inferred from mitochondrial genomes according to diverging time estimates, and the Chrysomeloidea family formed around 154.1 Mya in the Late Jurassic—although the majority of Cerambycidae subfamilies originated considerably earlier [11]. We found that they are largely congruent with the radiation of hosts from Cerambycidae (hosts of *Billaea*, Dexiinae) to Chrysomelidae (hosts of *Macquara*, Tachininae).

The mitochondrial genome exhibiting the characteristics of a small size, matrilineal inheritance, and high copy numbers has become a powerful marker for specimen identification, phylogenetic relationships, and comparative genomics analysis. To date, only two mitogenomes in this subfamily have been sequenced and assembled, and no mitogenomes of *Macquartia* have been released on the NCBI. The lack of mitogenomic data has limited our understanding of the evolution of Tachinidae. Here, we document the mitogenomic data of *Macquartia* and reconstruct the phylogenetic relationships of the family Tachinidae with newly sequenced mitogenomes to broaden our understanding of its mitogenomic structures and phylogenetic relationships. In addition, 287 specimens of *Macquartia* from China were examined, and 4 new species of *Macquartia* from China were discovered and described; a key to 12 species from China is given based on the examined material and related data. Hopefully, this study will promote future research into the taxonomy and evolutionary development of Tachinidae.

## 2. Materials and Methods

### 2.1. Sampling Collection and Identification

The collected information on tachinid specimens in this study is shown in (Table S1). Four new tachinids for molecular analysis were collected by malaise traps from the Dalaoling National Natural Reserve (31°4′35.6″ N and 110°56′11.6″ E), Hubei Province, China, in July, 2017 and 2018. All collected samples were preserved in 100% ethanol under −20 °C and deposited in the Museum of Beijing Forestry University, Beijing, China. Specimens of *Macquartia* were morphologically identified based upon morphological characteristics, especially male genitalia, by the corresponding author. The morphological terminology used in the descriptions follows McAlpine [12], except for the terms “phallus”, “pregonite”, and “postgonite” instead of “aedeagus”, “gonopod”, and “paramere”, respectively, which follow Sinclair [13]. Specimens were examined with a Leika 205A stereomicroscope. Measurements of heads followed Tschorsnig and Richter [10]. Dissections of male terminalia were carried out following the method described by O’Hara [14] and the dissected terminalia were placed in glycerine in a small plastic tube pinned together with the source specimen. Images were taken with a Leica 205A stereomicroscope (Leica, St. Gallen, Switzerland) equipped with a Leica CCD camera (Leica, St. Gallen, Switzerland) and images were blended with Leica Application Suiter Version 4.12.0.

### 2.2. DNA Extraction, Mitogenomes Sequencing, Assembly, and Annotation

Total genomic DNA was extracted from a single fly using the DNeasy Blood and Tissue kit (Qiagen, Hilden, Germany) following the manufacturer’s instructions for the total DNA purification of animal tissues. The little genomic DNA was used for the PCR amplification and sequencing to obtain DNA sequences of *cox1* [15]; subsequently, the remaining DNA was pooled and sequenced using the Illumina Novaseq 6000 (PE150, Illumina, San Diego, CA, USA) platform. The high-quality sequences were assembled using IDBA-1.1.1 [16] and acquired the best-fit mitochondrial scaffolds using BLAST searches above at least 98% [17] with *cox1* as bait sequences [18,19]. Protein-coding genes and ribosomal RNA genes were annotated by aligning with the homologous genes reported in other Calyptratae flies. The positions of 22 tRNAs were predicted using the MITOS Web Server (available at http://mitos2.bioinf.uni-leipzig.de/index.py) (accessed on 8 February 2022) [20]. The mitogenomes of *Macquartia* were uploaded to GenBank with the accession numbers of OL681848-OL681851.

### 2.3. Sequence Analysis

The circular mitogenome maps were depicted using OGDRAW (https://chlorobox.mpimp-golm.mpg.de/OGDraw.html) (accessed on 21 February 2022) [21]. The nucleotide composition and relative synonymous codon usage (RSCU) of these four mitogenomes were calculated with PhyloSuite [22]. The nucleotide compositional differences were calculated based on the AT-skew = (A − T)/(A + T) and GC-skew = (G − C)/(G + C) formulas [23]. The sliding window analysis (a sliding window of 200 bp in 20 bp overlapping steps) within DnaSP v6 was conducted to determine the nucleotide diversity (Pi) based on the concatenated alignments of 13 PCGs among four *Macquartia* mitogenomes. The ratios of Ka (nonsynonymous substitutions)/Ks (synonymous substitutions) based on 13 aligned protein-coding genes were estimated with DnaSP v6 [24]. MEGA 5 was used to estimate the average genetic distances within four *Macquartia* species under the Kimura 2-parameter mode [25].

### 2.4. Phylogenetic Analyses

Mitochondrial genomes of 19 species representing all four subfamilies of Tachinidae were selected for phylogeny reconstruction, including the four new mitogenomes documented in the present study. *Lucilia sericata* (Calliphoridae) and *Sarcophaga crassipalpis* (Sarcophagidae) were chosen as outgroups. Phylogenetic analysis was performed on the dataset of 13 PCGs, where all the assembled PCGs of 19 mitogenomes were aligned through MAFFT [26]. The optimal partitioning scheme and best models were selected by PartitionFinder 2 [27]. The Bayesian inference (BI) analyses were conducted through the online CIPRES Science Gateway [28]. The BI analyses were carried out through two independent Markov chain Monte Carlo (MCMC) chains, which were executed with 10 million iterations, with sampling every 1000 generations. The initial 25% samples were discarded as burn-in, while the remaining were used to form the consensus tree and posterior probability (PP) values. The convergence of the individual runs is assumed after the average standard deviation of split frequencies < 0.01 in MrBayes 3.2.6 and effective sample size (ESS) > 200 in Tracer [29,30].

## 3. Results and Discussion

### 3.1. Taxonomy

Genus *Macquartia* Robineau-Desvoidy, 1830.

*Macquartia* Robineau-Desvoidy, 1830: 204. Type species: *Macquartia rubripes* Robineau-Desvoidy, 1830 (by designation of Townsend, 1916).

**Remarks:** We follow Herting and Dely-Drskovits (1993) [7], Tschorsnig and Herting (1994) [9], Tschorsnig and Richter (1998) [10], Chao et al. (1998) [2], O’Hara Shima and Zhang (2009) [3], O’Hara, Henderson and Wood (2020) [1] in a broad definition of the genus *Macquartia*. For practical reasons, and to reduce the amount of changes in traditional binominal usages.

**Generic diagnosis.** *Macquartia* may be easily distinguished from other tachinids by the following characters: hairs or setulae on posteroventral half of head all black, without any pale hair. Second costal section with fine hairs ventrally. Scutum with three pairs each of presutural dorsocentral bristles and postsutural intra-alar bristles. Preapical posteroventral seta on hind tibia distinctly shorter than the preapical anteroventral seta.

**Description.** Body length more than 4.0 mm. Eye densely covered with hairs, each hair longer than combined diameter of three eye facets. Frons of male at most one-fourth eye width in dorsal view, proclinate orbital setae absent in male, or with two proclinate orbital setae in female. Ocellar setae developed, proclinate. Lower margin of face usually not visible in profile. Facial ridge with setae on lower half or less. Hairs or setulae on posteroventral half of head all black, without pale hairs. First flagellomere not pointed apically, less than two times as long as pedicel. Arista thickened on basal one-fourth or less, first aristomere distinctly shorter, arista short pubescent, the longest hairs longer than basal diameter of arista or plumose. Palpus dark brown or at least reddish yellow at apex. Scutellum and abdomen black. Scutum before suture without dark stripes, with two or three pairs of presutural and usually three postsutural dorsocentral setae, two or three postsutural intra-alar setae present. Scutellum usually with three pairs of setae along its margin, with strong crossed apical setae, but weaker than subapical scutellar setae, lateral scutellar setae absent or present. Prosternum and proepisternum bare. Postpronotum with two or three setae, arranged in a straight line. Katepisternum with two or three setae, anepimeral setae well developed, anatergite almost always with a group of minute hairs or setulae below lower calypter. Second costal section of wing with fine hairs ventrally. Only base of vein R_4+5_ with hairs. Wing cell r_4+5_ open or with a very short petiole, Bend of vein M without continuation or with a very short stub. Dorsal surface of lower calypter bare, divergent from scutellum or not divergent from scutellum. Legs usually black, sometimes reddish yellow. Inner anterior surface of fore coxa bare or predominantly bare. Preapicall posteroventral seta on hind tibia distinctly shorter than preapical anteroventral seta. Middorsal depression on abdominal syntergite 1 + 2 not or extending back to the hind margin of that segment, without or with two median marginal setae, fourth tergite barely with a complete row of discal setae. Male genitalia very long, middle part of cerci narrowed and pointed, surstyli more or less narrowed.

**Key to Chinese species of *Macquartia***

1 Middorsal excavation of abdominal syntergite 1 + 2 extending or nearly extending to its posterior margin. Three or four postsutural dorsocentral setae. Palpi reddish yellow.…………………………………………………………………………………….....2
  - Middorsal excavation of abdominal syntergite 1 + 2 not extending to its posterior margin. Three postsutural dorsocentral setae. Palpi dark brown or reddish yellow…………………………………………………………………………………..5

2 Parafacial bare, at most with 3–4 hairs below lowest frontal seta. Aristal hairs at least more than diameter of aristal base. Four or three postsutural dorsocentral setae. Two katepisternal setae. Lower calyptrae divergent from scutellum. Thoracic scutum with gray pruinosity, abdomen black, with sparse pruinosity or without pruinosity, syntergite 1 + 2 with a pair of lateral marginal seta, without median marginal seta; third tergite with two median marginal setae, fourth tergite with a row of marginal setae……………………………………………………………………………………….…3
  - Parafacial hairy. Arista almost bare; four postsutural dorsocentral setae; three katepisternal setae. Lower calyptrae not divergent from scutellum. Abdomen black, with gray pruinosity and markings; tergite three with large black marking………………………………………………………….*M. tessellum* Meigen

3 Pedicel and basal half of flagellomere dark brown. Four postsutural dorsocentral setae. Basicosta reddish yellow. Third tergite with two median discal setae in male and absent in female, fourth tergite with two to a row of discal setae in male and two setae in female, ocelli red …………………………………………………………...*M. pubiceps* Zetterstedt
  - Pedicel and basal half of flagellomere yellow. Three postsutural dorsocentral setae. Third and fouth tergites without median discal setae in both sexes, ocelli yellow.…………………………………………………………………………………4

4 Basicosta dark brown. Legs black. …………………*M. flavipedicel* Zhang and Li sp. nov.
  - Basicosta and legs reddish yellow. Eyes with densely long hairs in male and sparsely short hairs in female. ………………*M. flavifemorata* Zhang and Li sp. nov.

5 Mid tibia with one anterodorsal seta. Lower calyptrae divergent from scutellum……...……………………………………………………………………………6
  - Mid tibia with 2–5 anterodorsal setae. Abdominal third tergite with 2–4 marginal setae………………………………………………………………………………….....7

6 Parafacial bare, at most with 3–4 hairs below lowest frontal seta. Legs black. syntergite 1 + 2 without median marginal and with 1–2 lateral marginal setae ……………………………………………………………*M. chinensis* Zhang and Li sp. nov.
  - Parafacial hairy only on upper half or hairy on whole length ……………………………………………….………………*M. macularis* Villeneuve

7 Parafacial hairy on whole length …………………………………………..……………...8
  - Parafacial bare, at most hairy on upper half..…………………………...……….....9

8 Pedicel and legs black ………………………………………………..……*M. dispar* Fallén
  - Pedicel and tibiae at least reddish yellow. Femora at least reddish yellow on ventral apex. Two katepisternal setae. Frons width at least 1/4 of eye width, frontal vitta about twice as wide as parafrontalia. Prealar seta about as long as hind supra-alar seta in famale. Abdomen covered with dense grayish pruinosity and regular gleaming marking…………………………………………*M. viridana* R.-D.

9 Lower calyptrae not divergent from scutellum. Palpi reddish yellow, seldom dark at apex…………………………………………………………………………………………10
  - Lower calyptrae divergent from scutellum..………………………………………11

10 Hind tibia with three preapical dorsal setae, middle one weaker; three katepisternal setae. Frons 1/7–1/8 of eye width, frontal vitta narrower than parafrontalia. Parafacial hairy on upper half. Abdomen with sparse grayish pruinosity in male and female, or female black, without pruinosity, syntergite 1 + 2 with 2 median marginal and 1–3 lateral marginal setae in both sexes, third and fourth tergites separately with 2 median discal and 1–3 pairs of lateral discal setae in both sexes…………*M. tenebricosa* Meigen
  - Hind tibia with two preapical dorsal setae; syntergite 1 + 2 without median marginal seta. Abdomen of female covered with covered grayish pruinosity, with gleaming markings ………………………………………*M. chalconota* Meigen

11 Abdominal syntergite 1 + 2 with two strong median marginal setae in male and weak setae in female. fourth tergite with 2–4 median discal setae………….*M. nudigena* Mesnil
  - Abdominal syntergite 1 + 2 without median marginal setae in both sexes.…………….…………..………………*M. brunneisquama* Zhang and Li sp. nov.

***Macquartia brunneisquama* Zhang and Li sp. nov.**

Figure 1A, Figure 2A, Figure 3A, Figure 4A, Figure 5A, Figure 6A, Figure 7A and Figure 8A.

**Type material.** HOLOTYPE ♂: **China**, Hubei, Yichang, Dalaoling National Natural Reserve, 1690–1790 m, 31°4′35.6″ N, 110°56′11.6″ E, 11–27 July 2017, malaise traps (BFU = Beijing Forestry University). PARATYPES. 5♂, same as holotype. 1♀, Qinghai, Qilian Mountains, Zhamasi, 3050 m, 38.90° N, 99.59° E, 19 August 2019, Li JJ (SYNU = Shenyang Normal University).

**Diagnosis.** Male frons as wide as anterior ocellus, parafacial hairy at most on upper half, ocelli yellow. Palpi reddish yellow. Two presutural and three postsutural dorsocentral setae. Legs black, mid tibia with two anterodorsal setae. Lower calyptrae divergent from scutellum. Middorsal excavation of abdominal syntergite 1 + 2 not extending to its posterior margin, with a pair of lateral marginal setae; third tergite with two median marginal and two pairs of lateral marginal setae, without median discal seta.

**Description.** Body length 4.2–5.5 mm.

**Male.** Head black, with sparsely grayish white pruinosity on fronto-orbital plate, inner parafacial and face, but dense on outer parafacial; lower face, lower occiput and genal dilation with dark grayish pruinosity; frontal vitta dark brown; ocelli whitish yellow; lunule dark brown; upper occiput black. Pedicel dark brown to reddish brown; the basal half of first flagellomere reddish yellow, apical half of first flagellomere dark brown; palpus reddish yellow. Frons strongly narrowed above, at the narrowest point 1–1.2 times as wide as anterior ocellus, in profile 1.6–1.8 times as long as the face; frontal vitta almost linear in front of ocellar triangle and strongly widened anteriorly; parafacial nearly parallel-side, 1.2–1.5 times as wide as (slightly narrower in profile) first flagellomere; face weakly concave, very weakly carinate on upper median portion between base of antenna, lower portion very weakly but warped forward; gena approximately 1/4 in profile as high as eye height; occiput flatted, not bulged. Inner vertical setae hair-like, not different from postocular setae row; ocellar seta slender, about 2/7 as long as eye height; 8–9 frontal setae, 2 upper setae finer and shorter, lowest seta nearly with middle level of pedicel; fronto-orbital plate with dense hairs, parafacial almost bare, only with 2–3 hairs below lowest fronto-orbital seta; 8–10 long and strong subvibrissal setae. Base of antenna nearly level with middle of eye; antenna short, at most 3/5 as long as face; pedicel with a long seta which is nearly as long as the first flagellomere; first flagellomere about 2.2 times as long as wide and 2 times as long as pedicel; arista short pubescent, about 1.5 times as long as pedicel and first flagellomere together, thickened on basal 1/5–1/6, second aristomere as long as wide. Prementum 2–2.5 times as long as wide, about as long as genal height; palpus about 1.3 times as long as first flagellomere.

Thorax black in ground color, with very thin grayish pruinosity on postpronotal lobe, presutural area of scutum and proepimeron. Hairs black, rather sparse short and erect or suberect on scutum and scutellum, and dense on pleura; one 1 presutural and 1–2 postsutural acrostichal setae; 2 presutural and 3 postsutural dorsocentral setae; 2 postsutural intra-alar seta; prealar seta weak slender, as wide as the second supra-alar seta; 2 postpronotal setae; 2 pairs of reclinate and strong marginal scutellar setae; apical scutellar setae about twice as long as scutellum; a pair of discal scutellar setae, about as long as scutellum. Two katepisteral setae and anterior one weak; one upper anterior and a row of (4–6) posterior anepisternal setea; one weak anepisternal seta.

Wing hyaline, weakly tinged with pale brown, more strongly tinged on basal portion; tegula and basicosta dark brown; lower calypter divergent from scutellum, brown to dark, expect the basal brownish; halters yellow. Costal spine short, approximately 0.7 times the length of crossvein r-m, base of vein R_4+5_ with 2–3 fine setulae dorsally and ventrally. Relative lengths of costal sectors second, third, and fourth approximately as 1:4:1.3; vein M from dm-cu crossvein to its bend about 5.5 times the distance between the bend and wing hind margin.

Legs black or dark brown, pulvilli yellowish, claws and pulvilli nearly as long as or longer than fifth tarsomere. Fore tibia with 2–3 shorter anteriodorsal setae, preapical anterodorsal seta about as long as preapical dorsal seta; mid tibia with 2 anterodorsal seta, upper one short, 2 posterior setae, 1strong ventral seta; hind tibia with a row of irregular anterodorsal, 2 of them strong, 2posterodorsal, and 2ventral setae, upper one short; 1 preapical anterodorsal seta shorter than 1preapical dorsal seta.

Abdomen long ovate, without pruinosity. Middorsal excavation of syntergite 1 + 2 not extending to its posterior margin, with 1–2 lateral marginal seta, and with some lateral discal setae; third tergite with two strong median marginal setae and 1–3 lateral marginal setae, without discal seta; fourth tergite without discal seta; fifth tergite with a row of discal setae. Base of sternite five distinctly convex, the depth of V-shaped median cleft of sternite five short, about 2/9 of the sternite length, inner margin of posterior lobe slightly pointed at apex. Pregonite short and bluntly rounded, postgonite triangular, short and pointed at apex, basiphallus cylindric, distiphallus short triangular and membranous. In caudal view, cerci thick at basal 3/4 and apical 1/4 narrowed and point, surstylus very short and slightly bent outward. In a lateral view, cerci slender and its apex slightly bent backward, surstylus bluntly round at apex.

**Female.** Differing from male as follows: frons wide, vertex about 1/3 of head width; frontal vitta at middle about twice as wide as fronto-orbital plate; 6–8 frontal setae, and with 2 proclinate orbical setae; 1 upward prevertical seta, about as long as occellar, but weaker than orbical seta; inner vertical setae strong, distinct different from postocular setae row; outer vertical setae about 3/4 as long as inner vertical setae; lunule brown; pedicel and the basal of first flagellomere reddish yellow. Claws and pulvilli shorter than fifth tarsomere.

**Etymology.** The specific epithet is taken from a male character of this species, i.e., lower calypter brown. It is derived from the Latin adjective brunneus (=brown) and the Latin noun squama (=calypter).

**Distribution.** China (Hubei, Qinghai).

**Remarks.** The new taxon differs from other species of *Macquartia* in having a narrow male frons, as wide as anterior ocellus, parafacial usually bare, ocelli yellow. Palpi reddish yellow. Gena approximately 2/5 in profile as high as eye height; two presutural and three postsutural dorsocentral setae. Lower calyptrae yellowish, divergent from scutellum. Legs black, mid tibia with two anterodorsal setae. Middorsal excavation of abdominal syntergite 1 + 2 not extending to its posterior margin, third tergite with two median marginal and two pairs of lateral marginal setae, without median discal seta.

***Macquartia*** ***chinensis* Zhang and Li sp. nov.**

Figure 1B, Figure 2B, Figure 3B, Figure 4B, Figure 5B, Figure 6B, Figure 7B and Figure 8B

**Type material.** HOLOTYPE ♂, **China**, Hubei, Yichang, Dalaoling National Natural Reserve, 1690–1790 m, 31°4′35.6″ N, 110°56′11.6″ E, 11–27 July 2017, malaise traps (BFU). PARATYPES. 6♂2♀, same as holotype (BFU). 3♂, Liaoning, Qingyuan, Wandianzi, 600–720 m, 41.97° N, 115.29° E, 21 July 2016, Li XY, Liang HC. 1♀, Yunnan, Lijiang, Yulong Snow Mountains, High Mountain Botanical Garden, 2685–3107 m, 26.90° N, 100.17° E, 20 July 2017, Liang HC (SYNU).

**Diagnosis.** Frons of male 1/3 as wide as first flagellomere. Parafacial bare. Pedicel reddish yellow, three presutural and three postsutural dorsocentral setae. Lower calyptrae divergent from scutellum. Mid tibia with 1 anterodorsal seta. Middorsal excavation of syntergite 1 + 2 not extending to its posterior margin, without lateral marginal seta, third and fourth tergites without median discal seta.

**Description.** Body length 4.9–7.1 mm.

**Male.** Head black, with sparsely grayish white pruinosity on fronto-orbital plate, parafacial and face; lower face, lower occiput, and genal dilation with dark grayish pruinosity; frontal vitta dark brown; ocelli reddish yellow to red; lunule dark brown to yellowish brown; upper occiput black. Antenna reddish yellow, except for apical half of first flagellomere dark brown; palpus reddish yellow. Frons strongly narrowed above, at the narrowest point as wide as base of arista or anterior ocelus, in a profile 1.4–1.6 times as long as the face; frontal vitta almost linear in front of ocellar triangle and strongly widened anteriorly; parafacial nearly parallel-side, about twice as wide as the first flagellomere; face weakly concave, lower portion very weakly but warped forward; gena about 2/9 (about 1/8–1/4 in profile) as high as eye height; occiput flatted, not bulged. Inner vertical setae fine and long, hair-like, not different from postocular setae row; ocellar seta slender, fine and long about 0.2–0.23 times as long as eye height; 9–10 frontal setae, 3–4 upper setae finer and shorter, lowest seta nearly with middle level of pedicel; fronto-orbital plate with hairs, parafacial almost bare, only with 3–6 hairs below lowest fronto-orbital seta; a row of (9–10) subvibrissal setae, which at most about 1/2 as long as vibrissal. Base of antenna nearly level with the middle of the eye; antenna short, 0.57–0.64 times as long as face; pedicel with a slender seta which is nearly as long as first flagellomere; first flagellomere 2.5–2.7 times as long as wide and two times as long as pedicel; arista short pubescent, about 1.5 times as long as pedicel and first flagellomere together, thickened on basal 1/5–1/6, second aristomere 1.2 times as long as wide. Prementum about 2.5 times as long as wide, about as long as genal height; palpus about 1.5 times as long as first flagellomere.

Thorax black in ground color, with very thin grayish pruinosity on postpronotal lobe, presutural area of scutum and proepimeron. Hairs black, rather sparse short and erect or suberect on scutum and scutellum, and short on pleura; one presutural and one postsutural acrostichal setae; three presutural and three postsutural dorsocentral setae; two postsutural intra-alar seta; prealar seta about as long as notopleural, and slightly longer or as wide as second supra-alar seta; two postpronotal setae; two pairs of reclinate and strong marginal scutellar setae; apical scutellar setae crossed, about twice as long as scutellum; two discal scutellar setae. Two katepisteral setae and anterior one weak; one upper anterior and a row of (4–6) posterior anepisternal setea; 1 weak anepisternal setae; anatergite hairy.

Wing hyaline, weakly tinged with pale brown, more strongly tinged on basal portion; tegula and basicosta dark brown; lower calypter pale brownish, fringe yellow; halters yellow. Costal spine short, about 1/2 length of crossvein r-m, base of vein R_4+5_ with 2–4 fine setulae dorsally and ventrally, Relative lengths of the costal sectors second, third, and fourth approximately as 1:3.5:1.2; vein M from dm-cu crossvein to its bend about 4.5 times distance between the bend and wing hind margin. The end of M and R_4+5_ nearly closed at wing margin.

Legs black, pulvilli yellowish, claws and pulvilli slightly longer than fifth tarsomere. Fore tibia with 3 short anteriodorsal setae, upper 3–4 short and weak, preapical anterodorsal seta about as long as preapical dorsal seta; mid tibia with 1 anterodorsal seta, 2–3 posterior setae, 1 ventral seta; hind tibia with 2–3 anterodorsal, 2 posterodorsal and 2 ventral setae; 1 preapical anterodorsal seta shorter than 1 preapical dorsal seta.

Abdomen long ovate, without pruinosity. Middorsal excavation of syntergite 1 + 2 not extending to its posterior margin, without median marginal and lateral marginal seta, but with some lateral discal setae; third tergite with 2 median marginal setae and 1–2 lateral marginal setae, without discal seta; fourth tergite without discal seta; fifth tergite with 4–6 discal setae. Sternite 5 nearly triangular, the depth of V-shaped median cleft of sternite 5 about 1/3 of the sternite length, posterior lobe bluntly rounded apically. Pregonite short, postgonite thin and pointed, distiphallus sclerotized, thin and long. In caudal view, cerci and surstylus short and slightly pointed at apex. In lateral view, cerci short, distinctly bent backward, surstylus thick and long, and slightly rounded at apex.

**Female.** Differing from male as follows: frons wide, vertex about 1/3 of head width; frontal vitta at middle slightly wider than fronto-orbital plate; 5–6 frontal setae, and with 2 proclinate orbical setae; 1 outward prevertical seta, about as long as occellar, but weaker than orbical seta; inner vertical setae strong, outer vertical setae is 0.6–0.8 times as long as inner vertical setae; antenna reddish yellow, except for first flagellomere apex dark brown. Apical 2/3 of palpus usually inflated. Claws and pulvilli shorter than fifth tarsomere.

**Etymology.** The specific epithet is taken from the locality of this species, i.e., China.

**Distribution.** China (Hubei, Liaoning, Yunnan).

**Remarks.** This species is distinguished from the other species of this genus in having reddish yellow pedicel, three presutural and three postsutural dorsocentral setae, mid tibia with one anterodorsal setae; middorsal excavation of abdominal syntergite 1 + 2 not extending to its posterior margin, without lateral marginal setae, tergites 3 and 4 without median discal seta.

***Macquartia flavipedicel* Zhang and Li sp. nov.**

Figure 1C, Figure 2C, Figure 3C, Figure 4C, Figure 5C, Figure 6C, Figure 7C and Figure 8C.

**Type material.** HOLOTYPE ♂, **China**, Hubei, Yichang, Dalaoling National Natural Reserve, 1690–1790 m, 31°4′35.6″ N, 110°56′11.6″ E, 11–21 July 2018, malaise traps (BFU). PARATYPES. 18♂1♀, same as holotype (SYNU).

**Diagnosis.** Parafacial bare, at most with 3–4 hairs below lowest frontal seta. Ocelli whitish yellow. Aristal hairs at least more than a diameter of the aristal base. Palpi reddish yellow. Thoracic scutum with gray pruinosity, three postsutural dorsocentral setae, two katepisternal setae. Lower calyptrae divergent from scutellum. Middorsal excavation of syntergite 1 + 2 extending or nearly to its posterior margin, with a pair of lateral marginal setae.

**Description.** Body length 4.9–7.0 mm.

**Male.** Head black, with sparsely grayish white pruinosity on fronto-orbital plate, parafacial and face; lower face, lower occiput and genal dilation with dark grayish pruinosity; frontal vitta dark brown; ocelli whitish yellow; lunule yellow; upper occiput black. Antenna reddish yellow, except for the apical half of first flagellomere dark brown; palpus reddish yellow. Frons strongly narrowed above, at the narrowest point 1/2–2/3 times as wide as antenna, in a profile 1.3 times as long as the face; frontal vitta almost linear in front of ocellar triangle and strongly widened anteriorly; parafacial nearly parallel-side, 2.0–3.0 times as wide as (nearly as wide as in profile) the first flagellomere; face weakly concave, lower portion very weakly but warped forward; gena about 1/5 (about 1/4 in profile) as high as eye height; occiput flatted, not bulged. Inner vertical setae fine and long, hair-like, not different from postocular setae row; ocellar seta slender, fine and long, about as long as 2/9 of the eye height; 8–10 frontal setae, 2 upper setae finer and shorter, lowest seta nearly with middle level of pedicel; fronto-orbital plate with dense hairs, parafacial almost bare, only with 3–4 hairs below lowest fronto-orbital seta; 5–7 long and strong subvibrissal setae; postocular setae close to posterior eye margin, long and directed forward on upper 1/2. Base of antenna nearly level with middle of eye; antenna short, at most 2/3 as long as face; pedicel with a long seta which is nearly as long as the first flagellomere; first flagellomere about twice as wide and 1.5 times as long as pedicel; arista short pubescent, about 1.5 times as long as pedicel and first flagellomere together, thickened on basal 1/5–1/6, second aristomere as long as wide. Prementum 2–2.5 times as long as wide, about as long as genal height; palpus about 1.5 times as long as first flagellomere.

Thorax shining black in ground color, with very thin grayish pruinosity on postpronotal lobe, presutural area of scutum, and proepimeron. Hairs black, rather sparse short and erect or suberect on scutum and scutellum, and dense and short on pleura; 1 presutural and 1 postsutural acrostichal setae; 3 presutural and 3 postsutural dorsocentral setae; 2–3 postsutural intra-alar seta, if 3 setae present, anterior one weak; prealar seta weak or hair-like, distinctly shorter than first supra-alar seta; 2 pairs of reclinate and strong marginal scutellar setae; apical scutellar setae crossed, about twice as long as scutellum; a pair of discal scutellar setae near apex, about as long as scutellum. Two katepisteral setae and an anterior weak one; one upper anterior and a row of (4–6) posterior anepisternal setea; one weak anepisternal seta, thin and long.

Wing hyaline, weakly tinged with pale brown, more strongly tinged on basal portion; tegula and basicosta dark brown; lower calypter pale brownish, fringe yellowish; halters yellow. Costal spine short, about 1/2 length of crossvein r-m, base of vein R_4+5_ with 3–4 fine setulae dorsally and ventrally, relative lengths of costal sectors second, third, and fourth approximately as 1:2.6:1; vein M from dm-cu crossvein to its bend at approximately six times distance between the bend and wing hind margin.

Legs black or dark brown, pulvilli yellowish, claws and pulvilli nearly as long as or longer than the fifth tarsomere. Fore tibia with 2–5 anteriodorsal setae, upper 3–4 short and weak, preapical anterodorsal seta about as long as preapical dorsal seta, 1 posterodorsal; mid tibia with 1 anterodorsal seta, 2 posterior setae, 1 ventral seta; hind tibia with a row of irregular anterodorsal, 3 or 4 of them strong, 2 posterodorsal and 2–3 ventral setae; 1 preapical anterodorsal seta shorter than 1 preapical dorsal seta.

Abdomen long ovate, without pruinosity. Middorsal excavation of syntergite 1 + 2 nearly extending to its posterior margin, without median marginal seta and lateral marginal seta, but with some lateral discal setae; third tergite with 2 strong median marginal setae and 1–2 lateral marginal setae, without discal seta; fourth tergite without discal seta; fifth tergite separately with a row of discal setae. Sternite 5 nearly rectangular, the depth of V-shaped median cleft short is about 1/6 of the sternite, inner margin of posterior lobe pointed at apex. Pregonite broad, postgonite short, apex bent downward and pointed hook-like, distiphallus thick and flat at apex. In caudal view, apical half of cerci slightly thick and apex pointed, surstylus short and bluntly round at apex. In lateral view, cerci slender and its apex slightly bent backward, surstylus short and thick, bluntly rounded at apex.

**Female.** Differing from male as follows: frons wide, vertex 0.28–0.29 of head width; frontal vitta at middle slightly wider than fronto-orbital plate; 5–6 frontal setae, and with 2 proclinate orbical setae; 1 outward prevertical seta, about as long as occellar, but weaker than orbical seta; inner vertical setae strong, distinct different from postocular setae row; antenna reddish yellow, except for first flagellomere apex dark brown, claws and pulvilli shorter than fif5th tarsomere.

**Etymology.** The specific epithet is taken from a character of this species, i.e., pedicel and basal half of flagellomere 1 reddish yellow. It is derived from the Latin adjective *flavus* (=yellow) and the Latin noun *pedicul* (=pedicel = second antennal segment).

**Distribution.** China (Hubei).

**Remarks.** This new species resembles *M. pubiceps* but can be characterized by yellow pedicel and basal half of flagellomere, three postsutural dorsocentral setae, basicosta dark brown, legs black, third and fourth tergites without median discal seta in both sexes.

***Macquartia flavifemorata* Zhang and Li sp. nov.**

**Figure 1:**
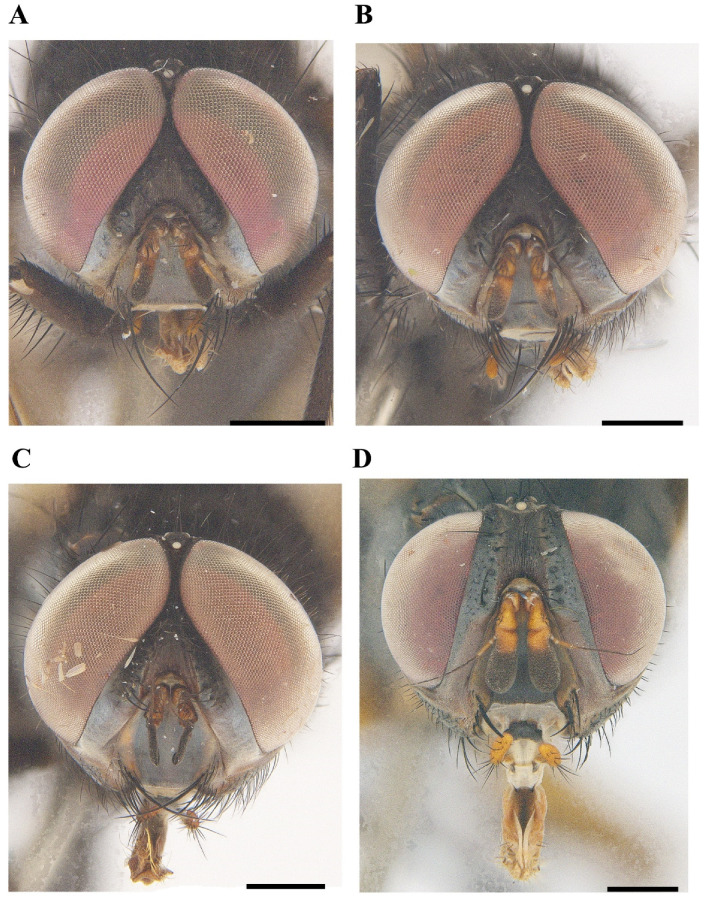
Anterior view of *Macquartia* spp. male heads. (**A**) *M. brunneisquama* sp. nov. (**B**) *M. chinensis* sp. nov. (**C**) *M. flavipedicel* sp. nov. (**D**) *M. flavifemorata* sp. nov. Scale bar = 0.5 mm.

**Figure 2:**
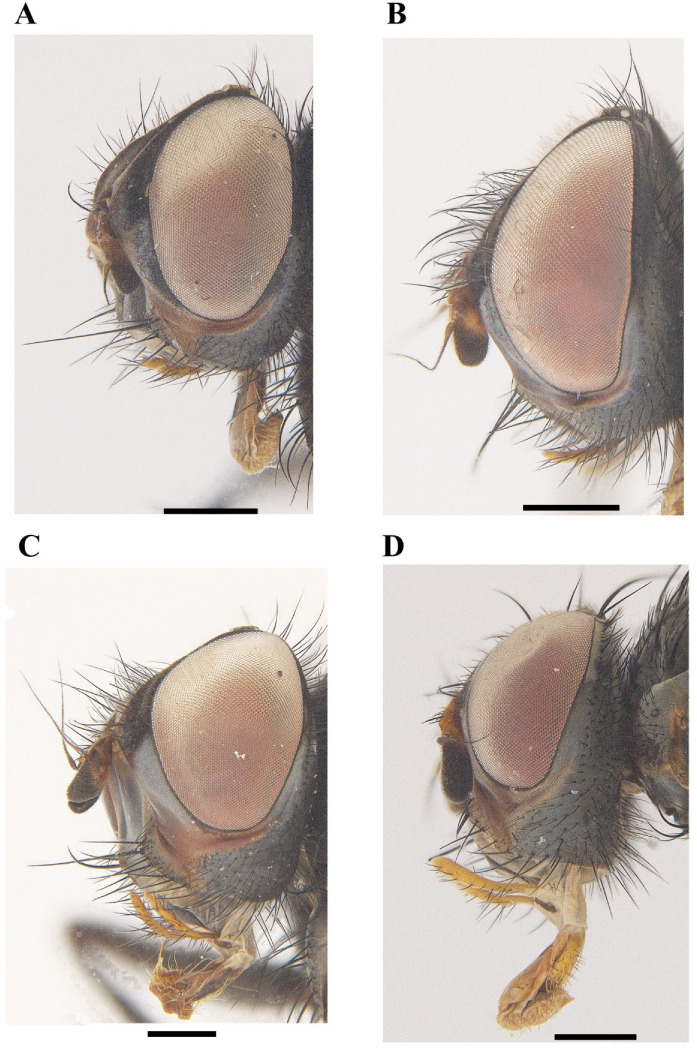
Lateral view of *Macquartia* spp. heads. (**A**) *M. brunneisquama* sp. nov. (**B**) *M. chinensis* sp. nov. (**C**) *M. flavipedicel* sp. nov. (**D**) *M. flavifemorata* sp. nov. Scale bar = 0.5 mm.

**Figure 3:**
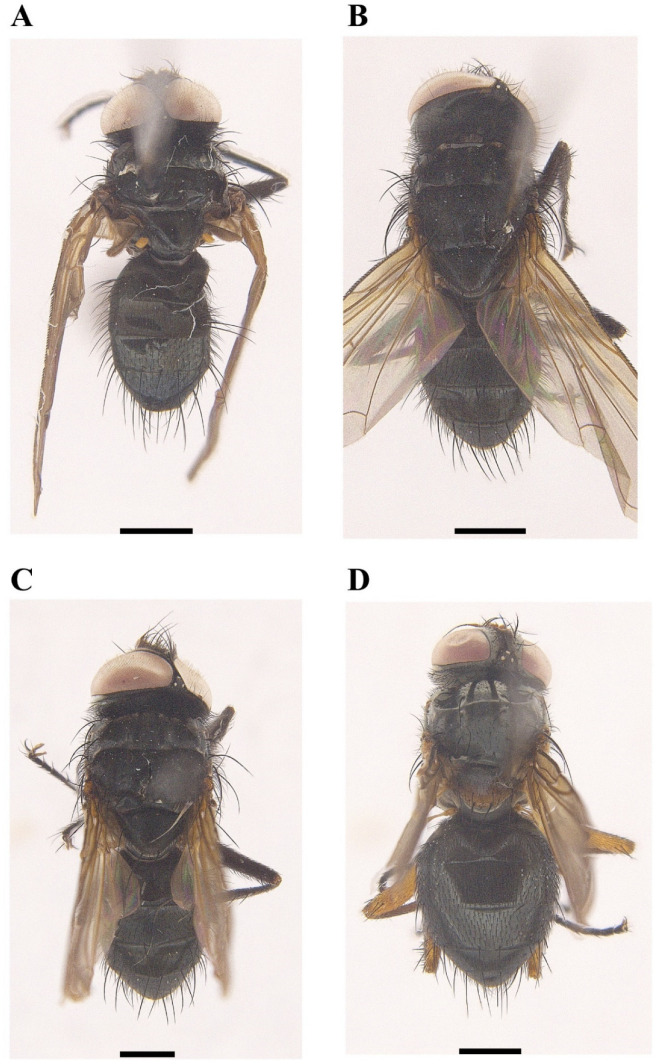
Dorsal view of *Macquartia* spp., male habitus. (**A**) *M. brunneisquama* sp. nov. (**B**) *M. chinensis* sp. nov. (**C**) *M. flavipedicel* sp. nov. (**D**) *M. flavifemorata* sp. nov. Scale bar = 1 mm.

**Figure 4:**
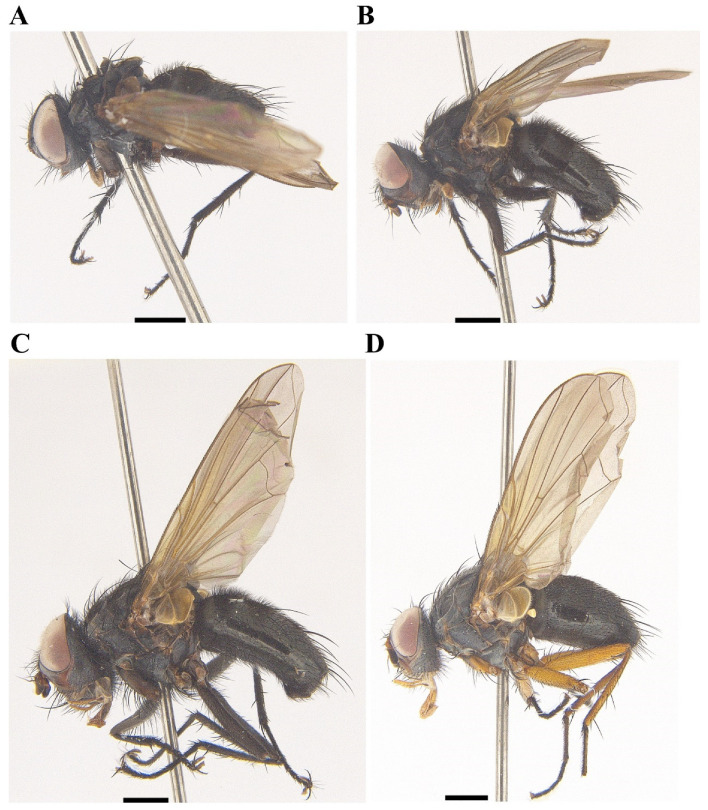
Lateral view of *Macquartia* spp., male habitus. (**A**) *M. brunneisquama* sp. nov. (**B**) *M. chinensis* sp. nov. (**C**) *M. flavipedicel* sp. nov. (**D**) *M. flavifemorata* sp. nov. Scale bar = 1 mm.

**Figure 6:**
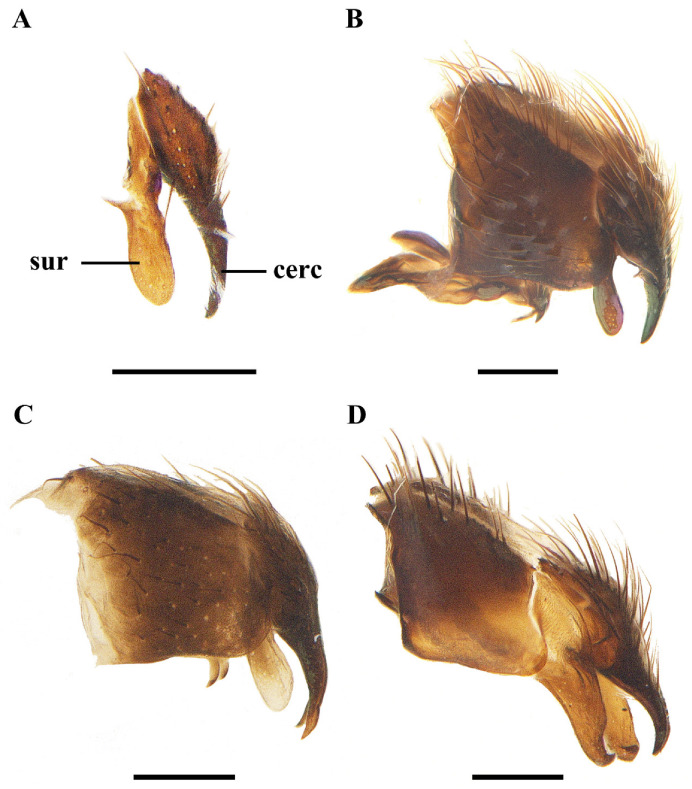
Lateral view of male terminalia of *Macquartia* spp. (**A**) *M. brunneisquama* sp. nov. (**B**) *M. chinensis* sp. nov. (**C**) *M. flavipedicel* sp. nov. (**D**) *M. flavifemorata* sp. nov. Scale bar = 0.2 mm. (Abbreviations: **cerc** = cercus; **sur** = surstylus.)

**Figure 7:**
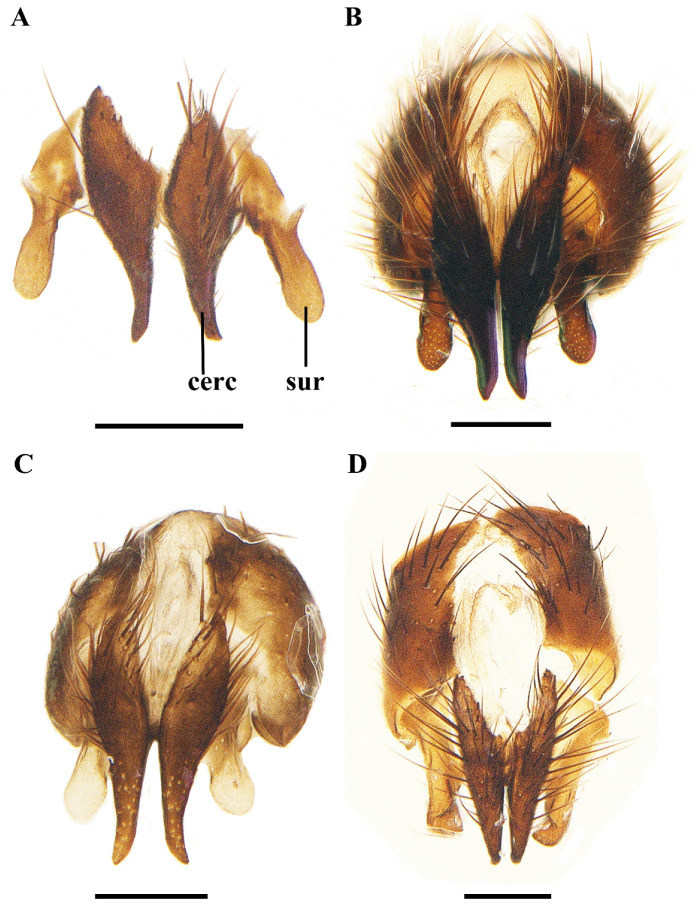
Caudal view of male terminalia of *Macquartia* spp. (**A**) *M. brunneisquama* sp. nov. (**B**) *M. chinensis* sp. nov. (**C**) *M. flavipedicel* sp. nov. (**D**) *M. flavifemorata* sp. nov. Scale bar = 0.2 mm. (Abbreviations: **cerc** = cercus; **sur** = surstylus.)

**Figure 8:**
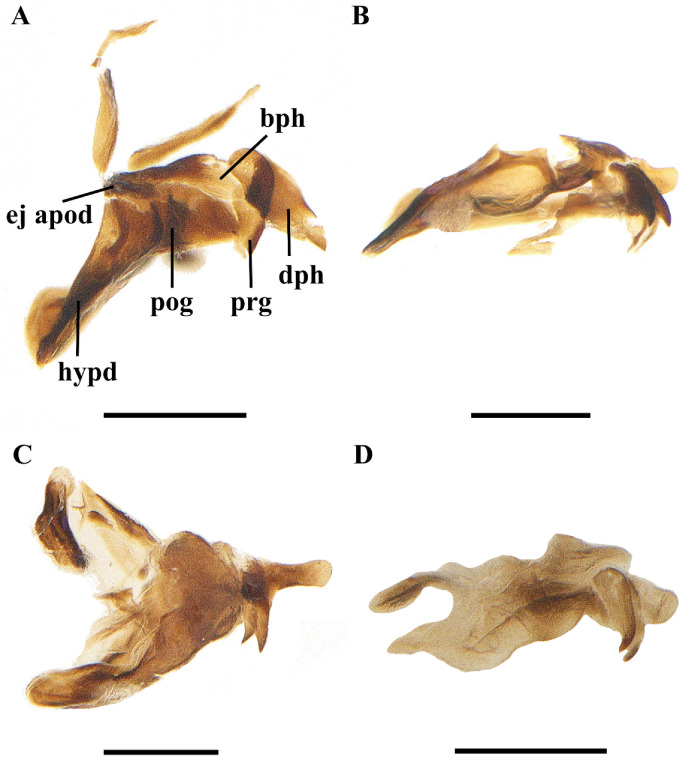
Lateral view of *Macquartia* spp. phallic complexes. (**A**) *M. brunneisquama* sp. nov. (**B**) *M. chinensis* sp. nov. (**C**) *M. flavipedicel* sp. nov. (**D**) *M. flavifemorata* sp. nov. Scale bars = 0.2 mm. (Abbreviations: **bph** = basiphallus; **dph** = distiphallus; **ej apod** = ejaculatory apodeme; **hypd** = hypandrium; **prg** = pregonite; **pog** = postgonite.)

Figure 1D, Figure 2D, Figure 3D, Figure 4D, Figure 6D, Figure 7D and Figure 8D.

**Type material.** HOLOTYPE ♀, **China**, Hubei, Yichang, Dalaoling National Natural Reserve, 1690–1790 m, 31°4′35.6″ N, 110°56′11.6″ E, 11–27 July 2017, malaise traps (BFU). PARATYPES. 1♂8♀, same as holotype (SYNU).

**Diagnosis.** Parafacial bare. Pedicel and basal half of flagellomere yellow, palpi, basicosta, and legs reddish yellow except dark tarsi, aristal hairs at least more than diameter of aristal base. Apical half of scutellum brown to yellowish. Two presutural and three postsutural dorsocentral setae, two katepisternal setae. Lower calyptrae divergent from scutellum. Middorsal excavation of syntergite 1 + 2 nearly to 4/5 of its posterior margin, third tergite with two weak median marginal setae in male, third and fourth tergites without median discal setae in both sexes.

**Description.** Resembles *Macquartia flavipedicel* sp. nov., but it is distinguished as follows:

Body length 4.0–6.5 mm.

**Male.** Frons strongly narrowed above, at the narrowest point 1/2–1/3 times as wide as antenna, in profile 1.2–1.5 times as long as face; parafacial about twice as wide as (about 1/2 as wide as in profile) first flagellomere; gena about 1/4 (about 1/4 in profile) as high as eye height; inner vertical setae fine and long, different from postocular setae row; ocellar seta slender, about 1/5 as long as eye height; first flagellomere about 2.5 times as long as wide and 2.2 times as long as pedicel. Thorax. With four dark longindinal thoracic vittae. Apical half of scutellum brown to yellowish. Two presutural and three postsutural dorsocentral setae; two postsutural intra-alar seta; without prealar seta; two supra-alar setae. Wing. Tegula dark brown; basicosta reddish yellow. Relative lengths of costal sectors second, third, and fourth approximately as 1:3.6:1.2; vein M from dm-cu crossvein to its bend about five times the distance between the bend and wing hind margin. Legs yellow, expect tarsi dark brown. Abdomen. Third tergite without median marginal seta; fifth tergite with two discal setae. Base of sternite 5 bluntly round, the depth of V-shaped median cleft of sternite 5 about 1/5 of the sternite length, posterior lobe bluntly rounded apical. Pregonite short and thick, postgonite short and pointed, distiphallus narrowed on apical half, and bluntly round at apex. In caudal view, cerci thick and short, apex bent backward, surstylus thick and apex slightly bent inward. In lateral view, cerci thick and its apex bent backward, surstylus thick and spoon-like, apex bent front-outward.

**Female.** Differing from male as follows: frons wide, vertex 0.27–0.3 of head width. Parafacial bare. Frontal vitta wider than fronto-orbital plate, latter with sparse black hairs. Seven frontal setae, one outward prevertical seta, two proclinate outer orbital setae, outer vertical seta about 1/2 length of inner vertical seta. Tegula brown at base and reddish at apical half, basicosta reddish yellow. Pulvilli yellowish, claws and pulvilli shorter than fifth tarsomere. Fore tibia with 2–3 anterodorsal setae, lower one longer, 1 preapical anterodorsal and 1 preapical dorsal seta in equal length, and 1 preapical posteroventral seta. Mid femur with 2–3 anterior setae and 2 preapical posterodorsal setae. Mid tibia with one anterodorsal, two posterior, and one anteroventral seta, apex with one anterior, one anterodorsal, one dorsal, one posterodorsal, one anteroventral, and one posteroventral seta. Hind femur with a complete row of anterodorsal setae, one anterior and three ventral (two at basal half, one at apex) setae. Hind tibia with a row of irregular anterodorsal setae, lower two of them strong, two posterodorsal and three anteroventral (lower two longer) setae, one preapical dorsal, one short anterodorsal and one preapical anteroventral seta. Abdomen without discal seta on intermediate tergites, syntergite 1 + 2 with 2–3 lateral marginal setae; third tergite with two median marginal setae or absent, with 2–3 lateral marginal setae; fifth tergite with a complete row of discal setae.

**Etymology.** The specific epithet is taken from a character of this species, i.e., legs reddish yellow. It is derived from the Latin adjective flavus (=yellow) and the Latin noun femur (=femora).

**Distribution.** China (Hubei).

**Remarks.** This new species is similar to *Macquartia flavipedicel* sp. nov., but differs from it having reddish yellow basicosta and legs. The major differences of four new species are listed (Table 1).

### 3.2. Mitogenome Organization

The four mitogenomes are closed circular molecules ranging from 16,617 to 18,159 bp, organized in the typical set of 37 genes (13 PCGs, 22 tRNA genes, and 2 rRNA genes) and a control region, respectively (Figure S1, Table S2). The gene arrangement of four new species is the same as that of ancestral insect mitogenomes [31]. Comparing the AT content within the whole mitogenome, the A + T-rich region were the highest while the PCGs were the lowest for all four species of *Macquartia* (Table S3). The four species almost all have negative AT-skews and positive GC-skews in 13PCGs, 22 tRNAs, and 2 rRNAs. The AT skew was congruent with that of several previously reported Oestroidea species [32,33].

Relative synonymous codon usage (RSCU) can directly indicate the preference of codon usage, and four *Macquartia* species exhibit the same codon preference pattern (Figure S2). The amino acid compositions showed the usage of synonymous codons with a higher frequency of AT than GC, demonstrating the strong AT bias in the whole mitochondrial genome. Inversely, some GC-rich codons are seldom utilized in *Macquartia* species. For example, the codon CGG is not used in *M. flavipedicel* sp. nov., and GCG is absent in *M. chinensis* sp. nov.

### 3.3. Nucleotide Diversity and Evolutionary Rate Analysis

**Figure 9:**
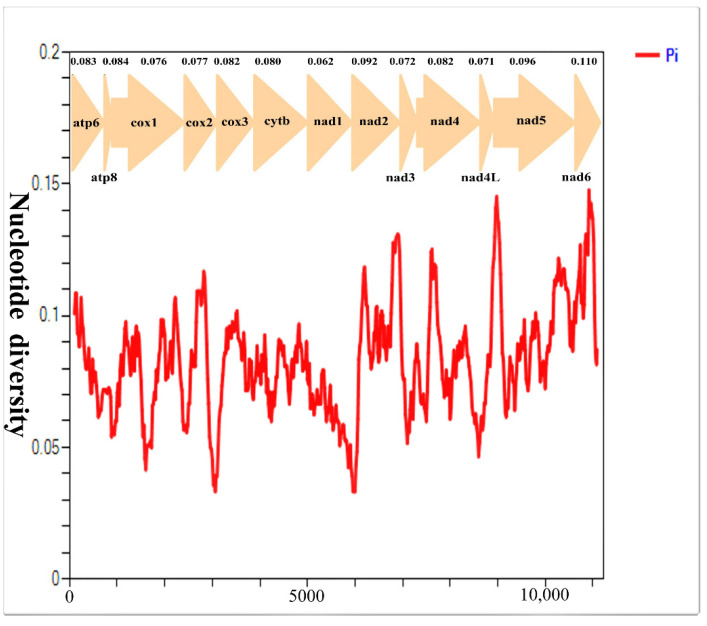
Nucleotide diversity on 13 PCGs in *Macquartia*. Sliding window analysis of 13 protein-coding genes among four *Macquartia* species. The red curve shows the value of Pi (nucleotide diversity). Pi value of each PCG is shown above the arrows.

**Figure 10:**
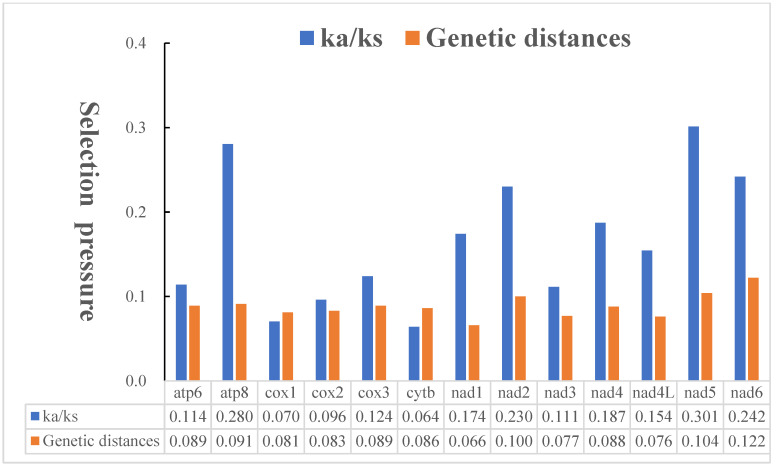
Genetic distances (on average) and ratio of nonsynonymous (Ka) to synonymous (Ks) substitution rates of each protein-coding gene among four *Macquartia* species, and the average value for each PCG is shown under the gene name.

The nucleotide diversity of the 13 PCG genes among our four tachinids is displayed in (Figure 9). The four with the distinctly highest variability were *nad6* (Pi = 0.110), *nad5* (Pi = 0.096), *nad2* (Pi = 0.092), and *atp8* (Pi = 0.084), while *nad1* (Pi = 0.062), *nad4L* (Pi = 0.071), *nad3* (Pi = 0.072), and *cox1* (Pi = 0.076) displayed comparatively low Pi values, indicating that they are the most conserved genes among the 13 PCGs. The genetic distance and Ka/Ks analyses also verge on virtually identical (Figure 10). The mean value of genetic distances within four mitogenomes shows that *nad6* (mean value = 0.122), *nad5* (0.104), and *nad2* (0.1) have undergone a relatively fast evolution. Conversely, *nad1* (0.066), *nad4L* (0.076), and *nad3* (0.077) with lower distances are evolving comparative slowly. The genes with the lowest and highest Ka were *cox1* (0.018) and *nad6* (0.065), respectively, while the lowest and highest Ks genes were *nad1* (0.172) and *cytb* (0.28), respectively (Figure S3). The average Ka/Ks analyses indicate that the values of the Ka/Ks ratio of 13 PCGs range from 0.064 to 0.301. This indicates that all mitochondrial PCGs are under a purifying selection (Ka/Ks < 1); therefore, they are appropriate for investigating phylogenetic relationships within the *Macquartia*. *Cytb* and *cox1*, with the lower value of the Ka/Ks ratio, experienced stronger purifying selection, which results in the *cox1* gene often being used as a DNA marker. On the contrary, the *nad5*, with the maximum value of 0.301, underwent its weakest purifying selection. In this study, we found that the *cox1* gene exhibits a relatively conserved and slow evolution rate compared to other PCGs, whereas *nad5* genes have a relatively faster evolution rate and evolve under comparative relaxed purifying selection, suggesting that *nad5* might be a potential marker for elucidating the intraspecific relationships for taxa with morphological features that are near and cryptic.

### 3.4. Phylogenetic Analysis

**Figure 11:**
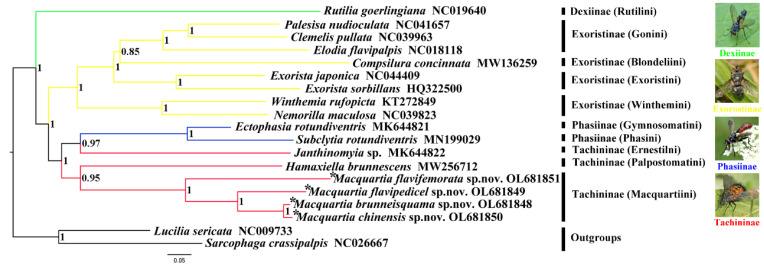
BI phylogenetic tree of nineteen species which consist of seventeen Tachinidae species and two outgroups. * Species documented in this study.

In this study, the mitogenomes of 4 new species and 15 available sequences on the GenBank were used to investigate the phylogenetic relationships of the Tachinidae. The tree topologies were reconstructed from BI analyses with high Bayesian posterior probability values (PP) in the most clades (Figure 11).

The phylogenetic relationship of this study shows similarities compared with those based on the morphology and molecular data revealed in previous studies [6]. The Tachinidae formed a monophyletic group with full support. The subfamily Tachininae is inferred to be paraphyletic, with the species *Janthinomyia* sp. as a sister group to Phasiinae. The monophyly of the three remaining subfamilies are confirmed, with the exception of Dexiinae, which is represented by only one species. The *Rutilia goerlingiana* established a distinct clade at the base of the Tachinidae, while we only used one tribe in our phylogenetic studies and the number of taxa sampled was too little to be typical. As a result, further data of Dexiinae mitogenomes are needed to validate the monophyly of this subfamily. More tachinid sequences are also required to examine the connections between Dexiinae and other subfamilies. Stireman et al. disagree with the subfamily-level associations established herein using mitogenomic data, which used nuclear genes for phylogeny reconstruction [6]. This inconsistency may be caused in part by the small number of taxa sampled here. Another factor might be the differing phylogenetic data that nuclear and mitochondrial genes possess, as suggested by Zhang et al. [34], Kutty et al. [35], and Yan et al. [36]. The tribe-level phylogenetic relationship of Exoristinae was (((Goniini + Blondeliini) + Exoristini) + Winthemiini), and the monophyly of each tribe is highly supported in this study (PP value > 0.8). The phylogenetic status of Exoristinae is established with higher supports on each node, which indicates that its phylogenetic relationship is reliable. The inter-genus relationships within Exoristinae are ((((*Palesisa* + *Clemelis*) + *Elodia* + *Compsilura*) + *Exorista*) + (*Winthemia* + *Nemorilla*)). The included *Macquartia* species were shown to form a monophyletic clade, with the tribe Palpostomatini occupying a sister position to the Macquartiini; moreover, the PP value (0.95) is very high, which strongly supports this topology structure. Our results show that all members (*M. brunneisquama*, *M. chinensis*, *M. flavifemorata*, and *M. flavipedicel*) are grouped into a clade with high support values (PP = 1). Within this group, *M. brunneisquama*, *M. chinensis*, and *M. flavipedicel* were clustered together and sisters to *M. flavifemorata*. We also found that *M. brunneisquama* and *M. chinensis* were placed into a subclade with strong nodal support values (PP = 1). As a consequence, this branch structure is still plausible and supports the conventional taxonomy. As only the mitogenomes of four species are detailed in this study, more species should be obtained to reveal the phylogenetic relationships of Tachinidae.

## 4. Conclusions

In this study, the combination of morphology and mitogenomes has allowed us to discover four new species of Tachinidae and infer its phylogenetic relationship. The four new species of *M. brunneisquama*, *M. chinensis*, *M. flavifemorata*, and *M. flavipedicel*, were described and illustrated and their comparison with congeners as well as the identification keys from China were provided. Then, we newly sequenced four complete mitogenomes to describe the characteristics of the genomes and align the phylogenetic relationships with their related taxa. This is the first report of mitogenome sequences from the genus *Macquartia* of the tribe Macquartiini. After our comparisons to other complete mitogenomes of Tachinidae, the species of *Macquartia* were found to be consistent in gene order, gene location, codon usage, nucleotide composition, and AT-biased pattern, and were highly conserved. Although the control regions vary greatly in length, their structure has not changed much, which includes four basic conservative regions. Bayesian inference phylogenetic analyses based on PCGs produce well-resolved topologies with most branches having strong support. These results offer a valuable framework for Tachinidae and could reinforce our understanding of the taxonomic status and phylogenetic relationships of Tachinidae. Additional taxon sampling will be necessary to verify the monophyly of Tachinidae and elucidate the relationships between Tachininae and other subfamilies and define the internal structure of this group.

## Figures and Tables

**Figure 5:**
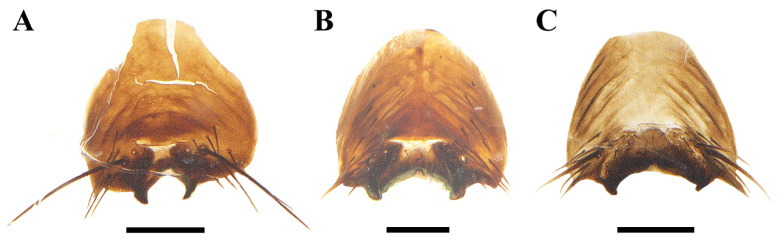
Ventral view of sternite 5 of *Macquartia* spp. (**A**) *M. brunneisquama* sp. nov. (**B**) *M. chinensis* sp. nov. (**C**) *M. flavipedicel* sp. nov. Scale bar = 0.2 mm.

**Table 1:** Comparison of four new species *of Macquartia* Robineau-Desvoidy.

	*M. brunneisquama* sp. nov.	*M. chinensis* sp. nov.	*M. flavipedicel* sp. nov.	*M. flavifemorata* sp. nov.
**Characters**	Parafacial hairy at most on upper half	Parafacial bare	Parafacial bare	Parafacial bare
	Pedicel dark to brown	Pedicel reddish yellow	Pedicel reddish yellow	Pedicel reddish yellow
	2 + 3 dorsocentral setae	3+3 dorsocentral setae	3 + 3 dorsocentral setae	2 + 3 dorsocentral setae
	Basicosta dark brown	Basicosta dark brown	Basicosta dark brown	Basicosta reddish yellow
	Legs dark brown	Legs dark brown	Legs dark brown	Legs reddish yellow
	Mid tibia with 2 anterodorsal setae	Mid tibia with 1 anterodorsal seta	Mid tibia with 1 anterodorsal seta	Mid tibia with 1 anterodorsal seta
	Middorsal excavation of abdominal syntergite 1 + 2 not extending to its posterior margin, with 1–2 lateral marginal seta	Middorsal excavation of syntergite 1 + 2 not extending to its posterior margin, without lateral marginal seta	Middorsal excavation of syntergite 1 + 2 extending or nearly to its posterior margin, with a pair of lateral marginal setae	Middorsal excavation of syntergite 1 + 2 nearly to 4/5 of its posterior margin, without lateral marginal setae

## Data Availability

The data presented in the study have been deposited in the NCBI database repository under the following accession numbers: OL681848, OL681849, OL681850, OL681851.

## Supplementary Materials

The following supporting information can be downloaded at: https://www.mdpi.com/article/10.3390/insects13121096/s1, Table S1. Information on collecting samples. Table S2. Annotations for the four species of *Macquartia*. Table S3. Nucleotide composition features of the four species of *Macquartia*. Figure S1. Circular maps of mitogenomes of four species in *Macquartia*. Figure S2. Relative synonymous codon usage (RSCU) in the mitogenomes of four *Macquartia* species. Figure S3. Synonymous and nonsynonymous substitutional rates of four *Macquartia* species for each PCG. Ks, synonymous substitutional rate; Ka, nonsynonymous substitutional rate.
