# Supplementary material for: Four New Species of Macquartia (Diptera: Oestroidea) from China and Phylogenetic Implications of Tachinidae
Source: Insects. 2022 Nov 28;13(12):1096. doi: 10.3390/insects13121096 (PMC9781235; doi:10.3390/insects13121096)
Supplement: Supplementary file 1 [file insects-13-01096-s001.zip › insects-1962159-supplementary.pdf]

**Supplementary**

**Table S1.** Information on collecting samples.

| Species | Specimen numbers | Collecting sites | Distribution |
|---|---|---|---|
| ***M. brunneisquama*** | 6♂ | Hubei | China (Hubei, Qinghai) |
| | 1♀ | Qinghai | |
| ***M. chinensis*** | 7♂2♀ | Hubei | China (Hubei, Liaoning, Yunnan) |
| | 3♂ | Liaoning | |
| | 1♀ | Yunnan | |
| ***M. flavipedicel*** | 19♂1♀ | Hubei | China (Hubei) |
| ***M. flavifemorata*** | 1♂9♀ | Hubei | China (Hubei) |
| ***M. dispar*** | 19♂ | Liaoning | Austria, Belgium, Britain, China (Liaoning, Ningxia, Qinghai), France, Hungary, Mongolia, Russia (East Siberia, North European Part), |
| | 1♂1♀ | Nei Mongolia | Sweden, Transcaucasus. |
| ***M. macularis*** | 1♀ | Liaoning | Albania, China (Liaoning, Ningxia, Shanxi, Sichuan), Mongolia, Morocco, Switzerland, Tunisia. |
| ***M. nudigena*** | 60♂11♀ | Liaoning | China (Liaoning, Nei Mongolia, Ningxia), Europe (northwards to Scotland, Sweden), Russia. |
| ***M. pubiceps*** | 1♀ | Anhui | China (Anhui, Fujian, Guizhou, Guangdong, Guangxi, Hainan, Hebei, Hubei, Liaoning, Nei Mongolia, Ningxia, Shaanxi, Shanxi), Europe (northwards to Scotland and Lapland), Japan, Russia, Transcaucasus. |
| | 1♀ | Fujian | |
| | 5♂ | Guangdong | |
| | 3♂1♀ | Guangxi | |
| | 5♂5♀ | Guizhou | |
| | 1♂2♀ | Hubei | |
| | 4♂2♀ | Liaoning | |
| | 2♂1♀ | Ningxia | |
| | 1♂3♀ | Shanxi | |
| ***M. tenebricosa*** | 13♂1♀ | Gansu | China (Beijing, Gansu, Hebei, Heilongjiang, Jilin, Liaoning, Nei Mongolia, Ningxia, Qinghai, Shanxi), Europe, Israel, Middle East, Mongolia, Russia, Transcaucasus. |
| | 2♂ | Hebei | |
| | 6♂ | Heilongjiang | |
| | 2♂ | Jilin | |
| | 52♂3♀ | Liaoning | |
| | 1♂ | Nei Mongolia | |
| | 7♂1♀ | Ningxia | |
| | 13♂ | Qinghai | |
| ***M. viridana*** | 5♂1♀ | Liaoning | China (Liaoning, Nei Mongolia, Zhejiang), Europe (Austria, Britain, France, Germany, Hungary, Spain, Switzerland), Russia. |

**Table S2.** Annotations for the four species of *Macquartia*.

| Gene | Direction | Size (bp) | Start Codons | Stop Codons | Anticodon | Intergenic Nucleotides |
|---|---|---|---|---|---|---|
| trnI | F | 66 | | | GAT | |
| trnQ | R | 69 | | | TTG | -5 |
| trnM | F | 66-68 | | | CAT | 3-5 |
| nad2 | F | 1017 | ATT | TAA | | 0 |
| trnW | F | 66 | | | TCA | -2 |
| trnC | R | 62-64 | | | GCA | -8 |
| trnY | R | 64 | | | GTA | 0 |
| cox1 | F | 1539 | TCG | TAA | | -2 |
| trnL2 | F | 66 | | | TAA | -5 |
| cox2 | F | 688 | ATG | T | | 4 |
| trnK | F | 71 | | | CTT | 0 |
| trnD | F | 65-66 | | | GTC | -1-0 |
| atp8 | F | 162 | ATT | TAA | | 0 |
| atp6 | F | 678 | ATG | TAA | | -7 |
| cox3 | F | 789 | ATG | TAA | | -1 |
| trnG | F | 63 | | | TCC | 6 |
| nad3 | F | 354 | ATC | TAG | | 0 |
| trnA | F | 64 | | | TGC | -2 |
| trnR | F | 62-63 | | | TCG | -1 |
| trnN | F | 64-65 | | | GTT | 0 |
| trnS1 | F | 67-68 | | | GCT | 0 |
| trnE | F | 63-64 | | | TTC | 2-3 |
| trnF | R | 64-65 | | | GAA | 18 |
| nad5 | R | 1735 | ATT | T | | 0 |
| trnH | R | 63-64 | | | GTG | 0 |
| nad4 | R | 1339 | ATG | T | | 0 |
| nad4l | R | 294 | ATG | TAA | | -7 |
| trnT | F | 63 | | | TGT | 2 |
| trnP | R | 66 | | | TGG | 0 |
| nad6 | F | 525 | ATT | TAA | | 2 |
| cob | F | 1137 | ATG | TAG | | -1 |
| trnS2 | F | 66-67 | | | TGA | -2 |
| nad1 | R | 939 | ATA | TAA | | 15 |
| trnL1 | R | 65 | | | TAG | 10 |
| rrnL | R | 1312-1316 | | | | 2 |
| trnV | R | 71 | | | TAC | 0 |
| rrnS | R | 783-811 | | | | -1 |

**Table S3.** Nucleotide composition features of the four species of *Macquartia*.

| Species | Regions | T(U) | C | A | G | A+T(%) | G+C(%) | AT-Skew | GC-Skew |
|---|---|---|---|---|---|---|---|---|---|
| *Macquartia brunneisquama* sp. nov. | Whole genome | 38.1 | 12.3 | 40.8 | 8.7 | 79.0 | 21.0 | 0.034 | -0.170 |
| | PCGs | 44.0 | 11.5 | 32.8 | 11.7 | 76.8 | 23.2 | -0.145 | 0.010 |
| | 1st codon | 42.7 | 9.4 | 37.2 | 10.7 | 79.9 | 20.1 | -0.070 | 0.061 |
| | 2nd codon | 43.5 | 12.7 | 29.9 | 13.8 | 73.4 | 26.6 | -0.185 | 0.041 |
| | 3rd codon | 45.6 | 12.3 | 31.4 | 10.7 | 77.0 | 23.0 | -0.185 | -0.070 |
| | tRNAs | 38.9 | 9.6 | 38.7 | 12.8 | 77.6 | 22.4 | -0.002 | 0.143 |
| | rRNAs | 42.4 | 6.4 | 39.2 | 12.0 | 81.6 | 18.4 | -0.040 | 0.304 |
| | Control region | 42.4 | 6.3 | 45.5 | 5.8 | 87.9 | 12.1 | 0.035 | -0.044 |
| *Macquartia chinensis* sp. nov. | Whole genome | 38.1 | 12.3 | 40.8 | 8.7 | 79.0 | 21.0 | 0.034 | -0.170 |
| | PCGs | 44.0 | 11.4 | 32.7 | 11.8 | 76.7 | 23.3 | -0.147 | 0.016 |
| | 1st codon | 42.8 | 9.4 | 37.2 | 10.6 | 79.9 | 20.1 | -0.070 | 0.061 |
| | 2nd codon | 43.6 | 12.7 | 29.8 | 13.9 | 73.4 | 26.6 | -0.187 | 0.044 |
| | 3rd codon | 45.7 | 12.2 | 31.2 | 10.9 | 76.9 | 23.1 | -0.189 | -0.057 |
| | tRNAs | 39.0 | 9.5 | 38.7 | 12.8 | 77.7 | 22.3 | -0.004 | 0.146 |
| | rRNAs | 42.8 | 6.3 | 39.1 | 11.9 | 81.9 | 18.1 | -0.045 | 0.307 |
| | Control region | 40.5 | 6.2 | 47.8 | 5.5 | 88.4 | 11.6 | 0.082 | -0.061 |
| *Macquartia flavifemorata* sp. nov. | Whole genome | 38.6 | 12.4 | 40.3 | 8.7 | 78.9 | 21.1 | 0.022 | -0.177 |
| | PCGs | 44.0 | 11.3 | 32.8 | 11.8 | 76.8 | 23.2 | -0.146 | 0.022 |
| | 1st codon | 42.7 | 9.3 | 37.3 | 10.8 | 80.0 | 20.0 | -0.068 | 0.074 |
| | 2nd codon | 43.5 | 12.6 | 29.9 | 14.1 | 73.4 | 26.6 | -0.186 | 0.056 |
| | 3rd codon | 45.8 | 12.2 | 31.4 | 10.7 | 77.1 | 22.9 | -0.187 | -0.063 |
| | tRNAs | 38.8 | 10.4 | 38.8 | 11.9 | 77.6 | 22.4 | 0.000 | 0.068 |
| | rRNAs | 42.6 | 6.1 | 39.7 | 11.6 | 82.3 | 17.7 | -0.035 | 0.312 |
| | Control region | 43.8 | 6.3 | 45.4 | 4.5 | 89.2 | 10.8 | 0.018 | -0.174 |
| *Macquartia flavipedicel* sp. nov. | Whole genome | 38.1 | 12.5 | 41.0 | 8.3 | 79.1 | 20.9 | 0.037 | -0.203 |
| | PCGs | 43.6 | 12.0 | 32.6 | 11.9 | 76.2 | 23.8 | -0.144 | -0.003 |
| | 1st codon | 42.0 | 10.3 | 37.0 | 10.7 | 79.0 | 21.0 | -0.064 | 0.020 |
| | 2nd codon | 43.1 | 13.1 | 29.6 | 14.2 | 72.7 | 27.3 | -0.185 | 0.041 |
| | 3rd codon | 45.5 | 12.5 | 31.2 | 10.8 | 76.7 | 23.3 | -0.187 | -0.075 |
| | tRNAs | 38.3 | 10.0 | 38.6 | 13.2 | 76.9 | 23.1 | 0.005 | 0.139 |
| | rRNAs | 42.6 | 6.5 | 38.6 | 12.3 | 81.2 | 18.8 | -0.050 | 0.310 |
| | Control region | 41.7 | 7.7 | 46.6 | 4.0 | 88.3 | 11.7 | 0.055 | -0.322 |

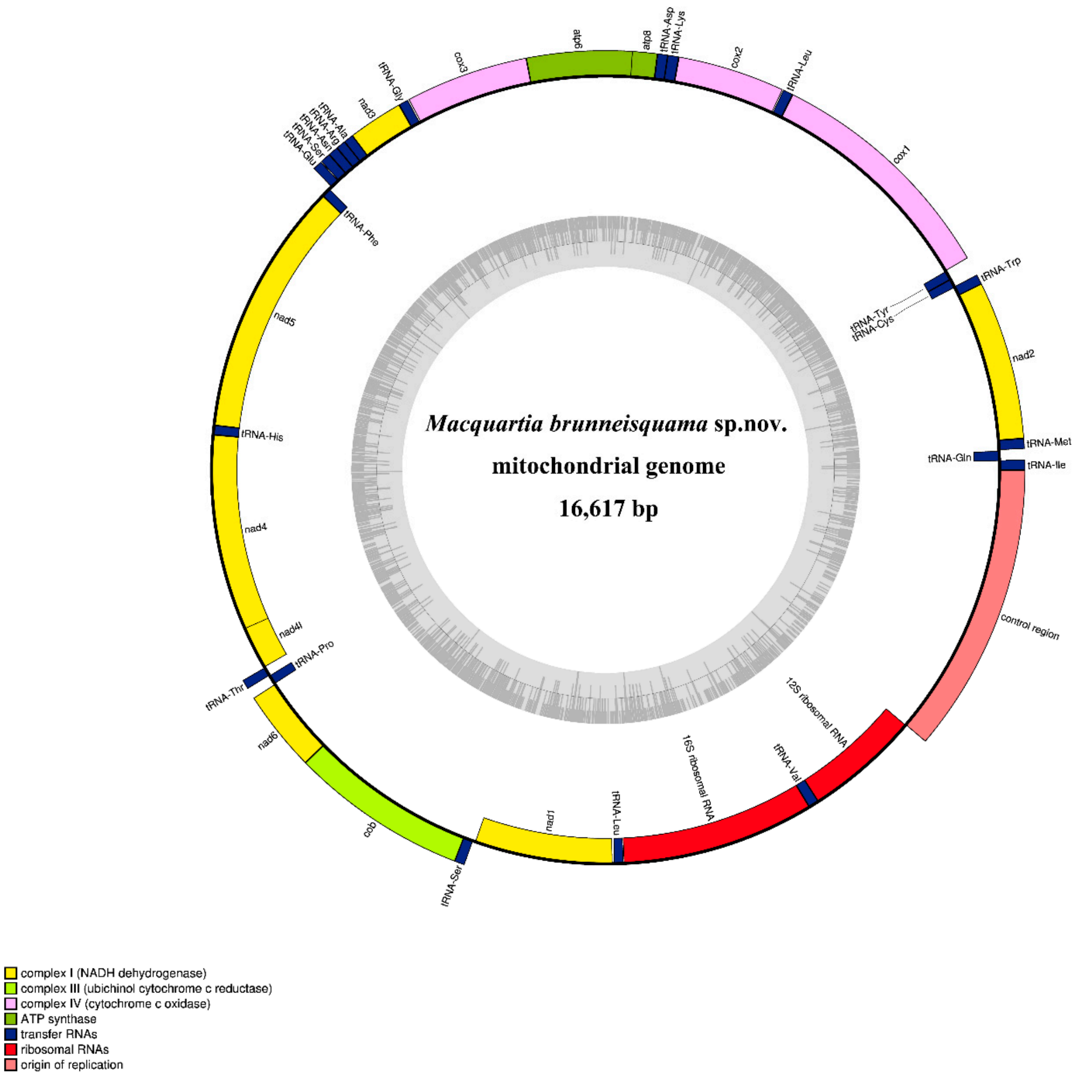

*Macquartia brunneisquama* sp.nov.
mitochondrial genome
16,617 bp
cox3
atp6
atp8
tRNA-Asp
tRNA-Lys
cox2
tRNA-Leu
cox1
tRNA-Trp
tRNA-Tyr
tRNA-Cys
nad2
tRNA-Met
tRNA-Gln
tRNA-Ile
control region
12S ribosomal RNA
tRNA-Val
16S ribosomal RNA
tRNA-Leu
nad1
tRNA-Ser
cob
nad6
tRNA-Thr
tRNA-Pro
nad4l
nad4
tRNA-His
nad5
tRNA-Phe
tRNA-Glu
tRNA-Ser
tRNA-Asn
tRNA-Arg
tRNA-Ala
nad3
tRNA-Gly
complex I (NADH dehydrogenase)
complex III (ubichinol cytochrome c reductase)
complex IV (cytochrome c oxidase)
ATP synthase
transfer RNAs
ribosomal RNAs
origin of replication

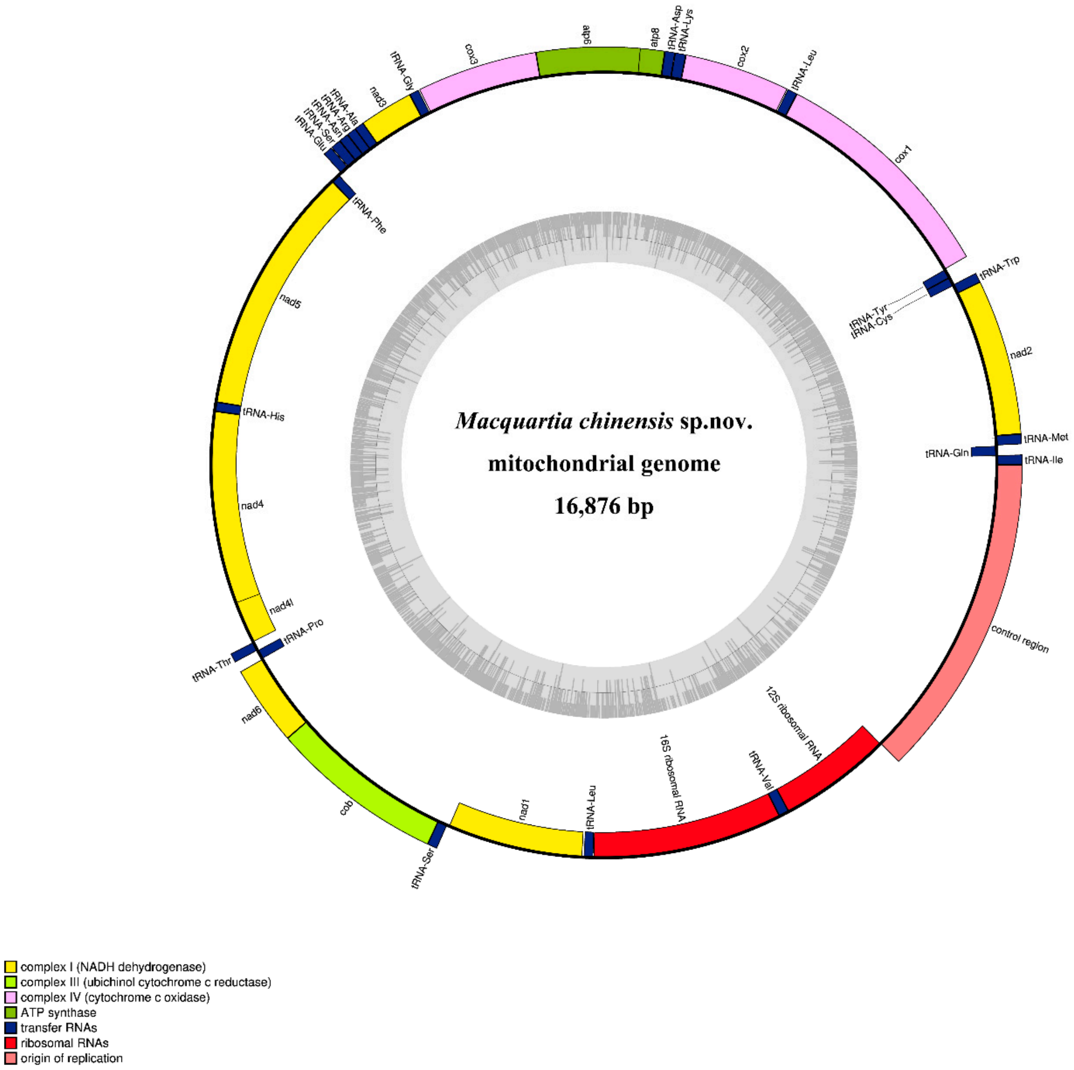
Macquartia chinensis sp.nov.
mitochondrial genome
16,876 bp
atp6
atp8
tRNA-Asp
tRNA-Lys
cox2
tRNA-Leu
cox1
tRNA-Trp
tRNA-Tyr
tRNA-Cys
nad2
tRNA-Met
tRNA-Gln
tRNA-Ile
control region
12S ribosomal RNA
tRNA-Val
16S ribosomal RNA
tRNA-Leu
nad1
tRNA-Ser
cob
nad6
tRNA-Thr
tRNA-Pro
nad4l
nad4
tRNA-His
nad5
tRNA-Phe
tRNA-Glu
tRNA-Ser
tRNA-Asn
tRNA-Arg
tRNA-Ala
nad3
tRNA-Gly
cox3
complex I (NADH dehydrogenase)
complex III (ubichinol cytochrome c reductase)
complex IV (cytochrome c oxidase)
ATP synthase
transfer RNAs
ribosomal RNAs
origin of replication

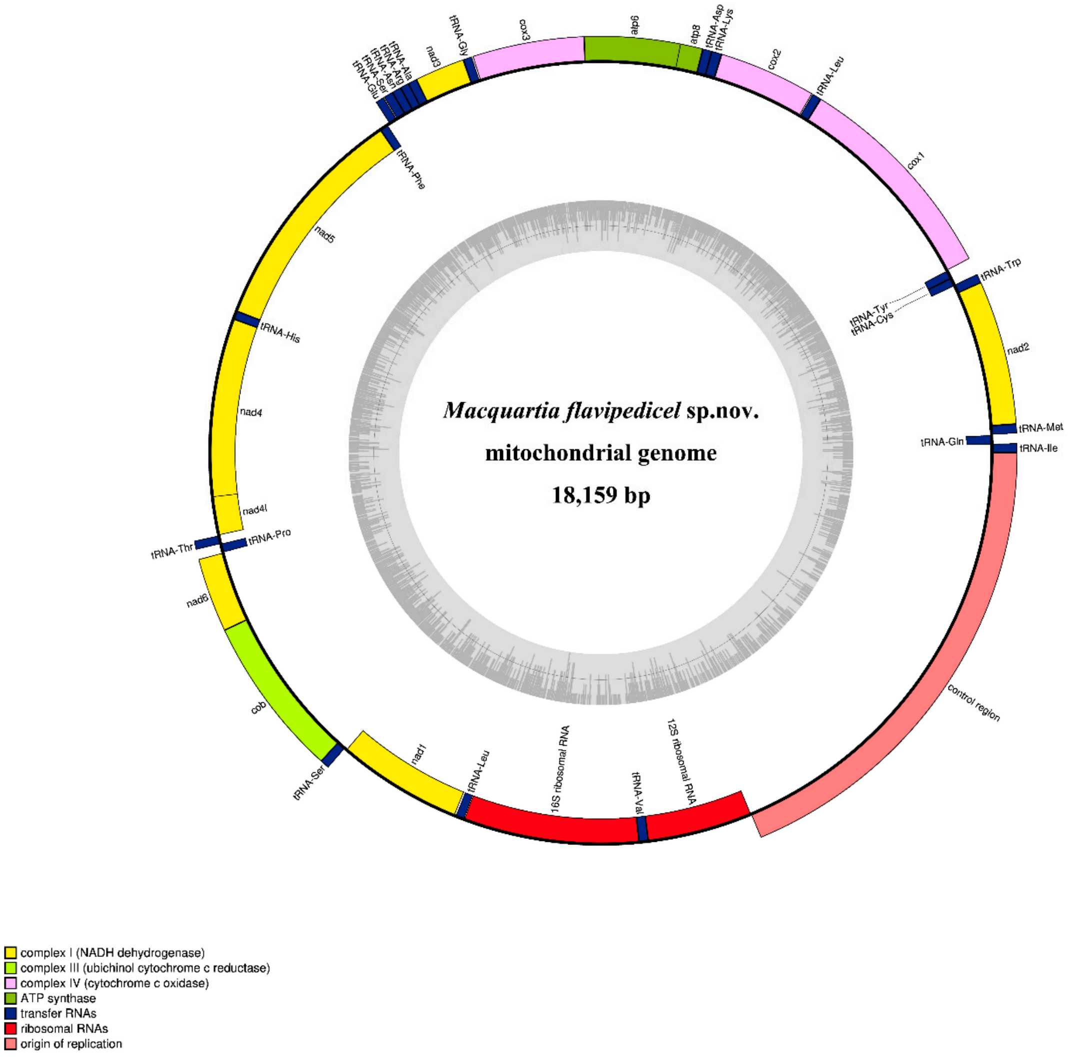
Macquartia flavipedicel sp.nov.
mitochondrial genome
18,159 bp
tRNA-Glu
tRNA-Ser
tRNA-Asn
tRNA-Arg
tRNA-Ala
nad3
tRNA-Gly
cox3
atp6
atp8
tRNA-Asp
tRNA-Lys
cox2
tRNA-Leu
cox1
tRNA-Trp
tRNA-Tyr
tRNA-Cys
nad2
tRNA-Met
tRNA-Gln
tRNA-Ile
control region
12S ribosomal RNA
tRNA-Val
16S ribosomal RNA
tRNA-Leu
nad1
tRNA-Ser
cob
nad6
tRNA-Thr
tRNA-Pro
nad4l
nad4
tRNA-His
nad5
tRNA-Phe
complex I (NADH dehydrogenase)
complex III (ubichinol cytochrome c reductase)
complex IV (cytochrome c oxidase)
ATP synthase
transfer RNAs
ribosomal RNAs
origin of replication

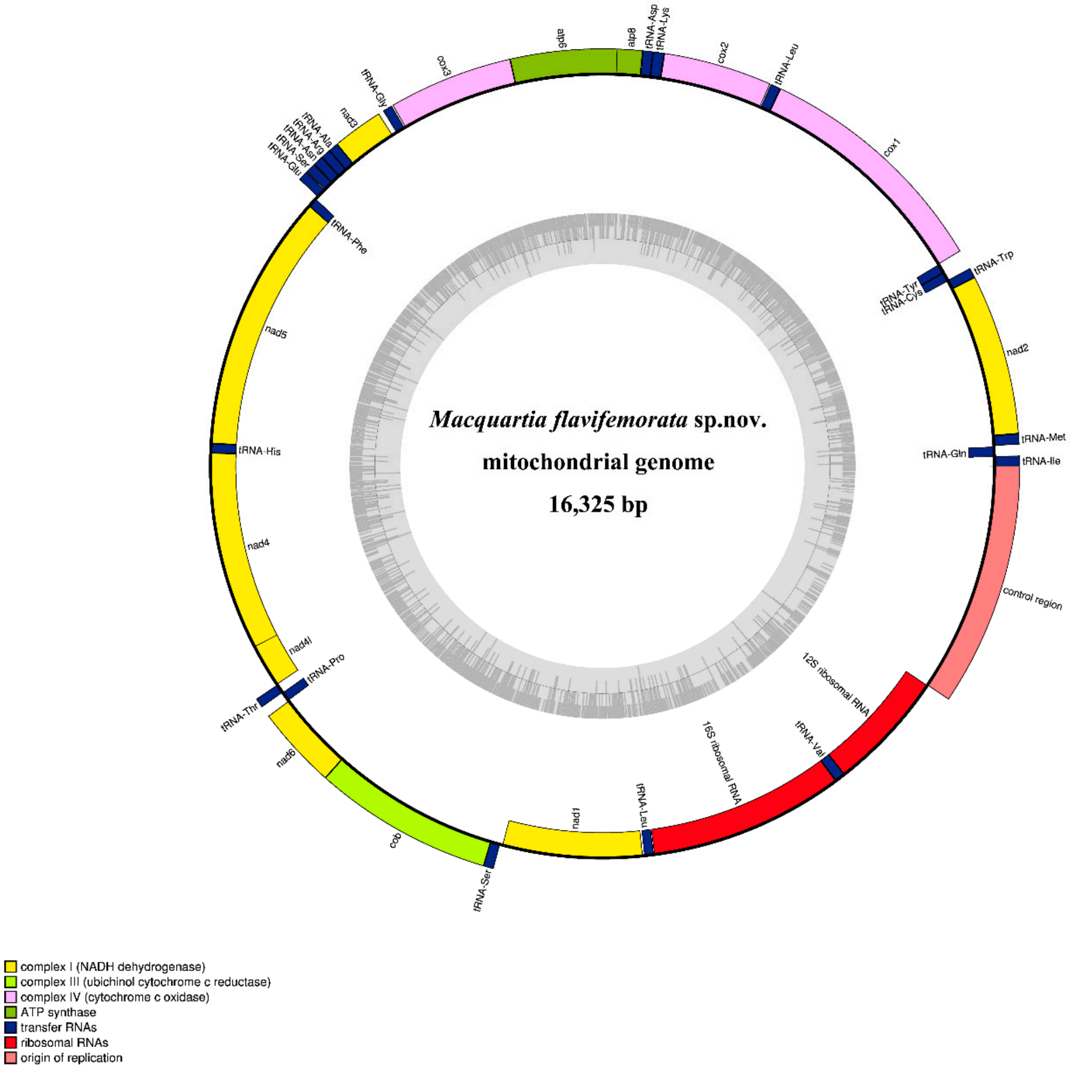


**Figure S1.** Circular maps of mitogenomes of four species in *Macquartia*.

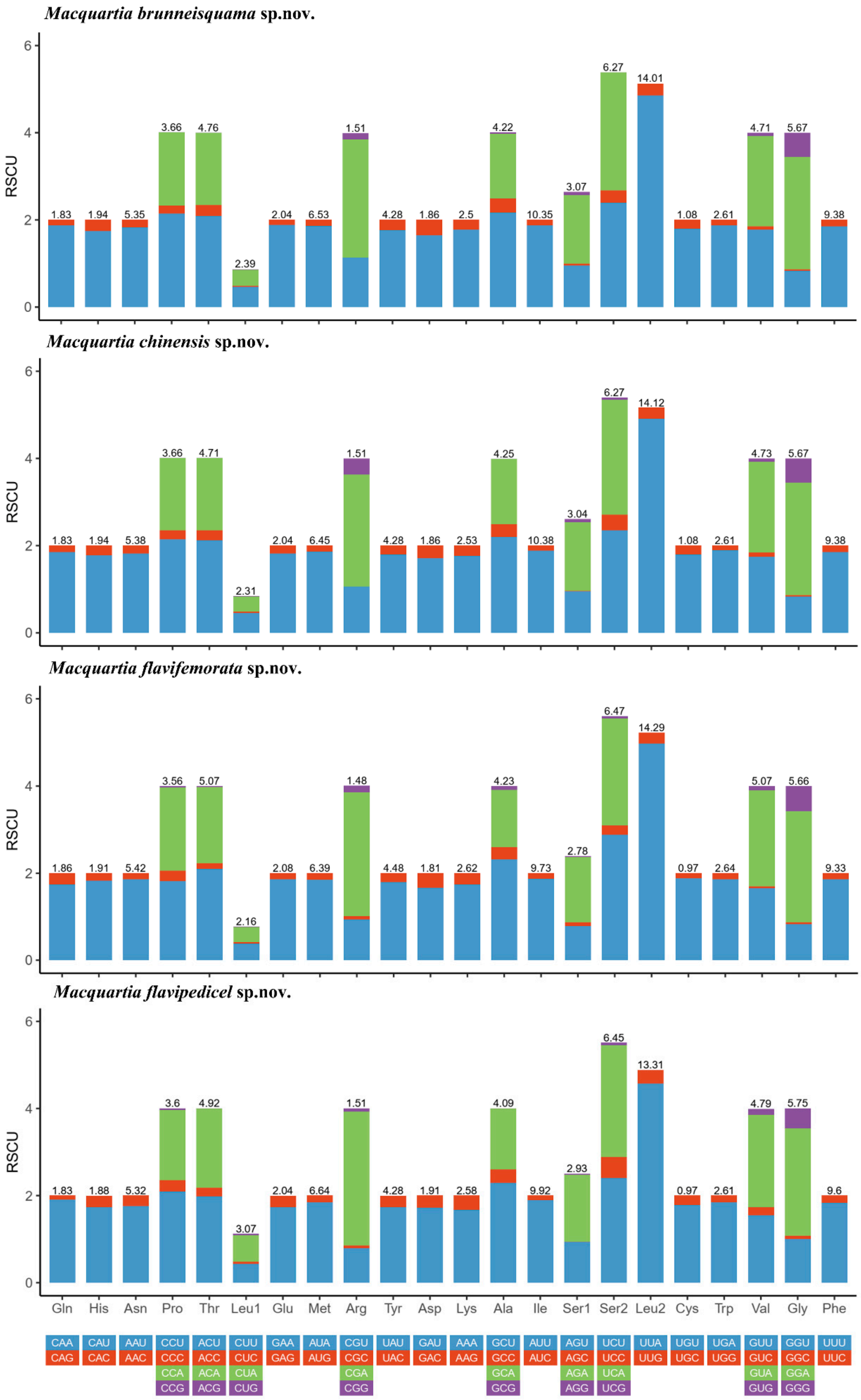


**Figure S2.** Relative synonymous codon usage (RSCU) in the mitogenomes of four *Macquartia* species.

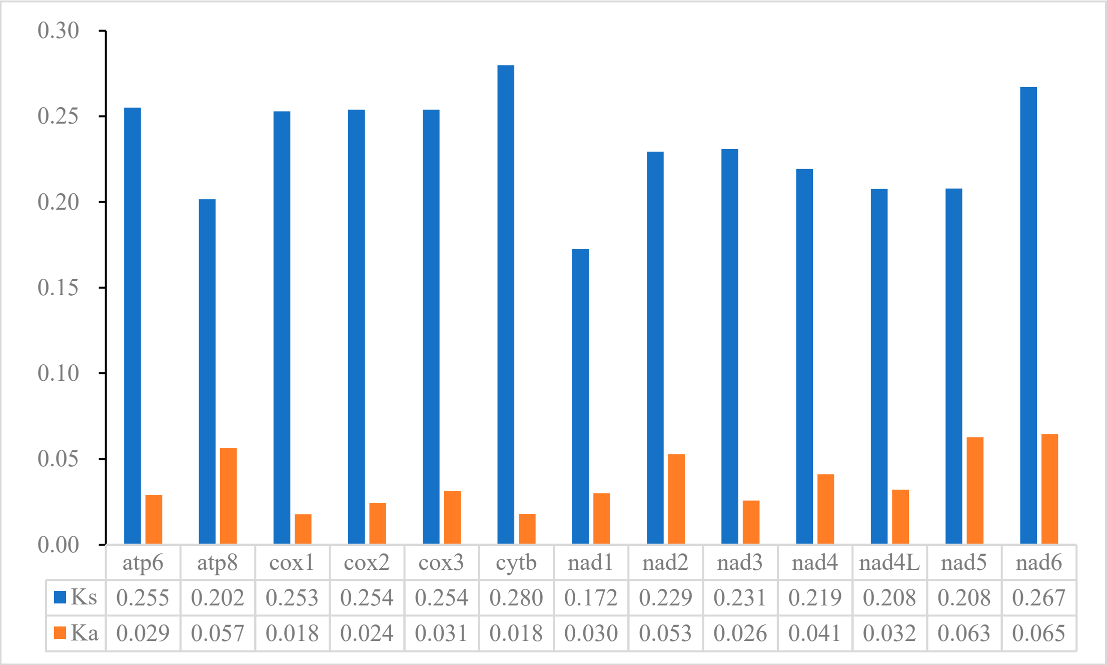


| | atp6 | atp8 | cox1 | cox2 | cox3 | cytb | nad1 | nad2 | nad3 | nad4 | nad4L | nad5 | nad6 |
|---|---|---|---|---|---|---|---|---|---|---|---|---|---|
| Ks | 0.255 | 0.202 | 0.253 | 0.254 | 0.254 | 0.280 | 0.172 | 0.229 | 0.231 | 0.219 | 0.208 | 0.208 | 0.267 |
| Ka | 0.029 | 0.057 | 0.018 | 0.024 | 0.031 | 0.018 | 0.030 | 0.053 | 0.026 | 0.041 | 0.032 | 0.063 | 0.065 |

**Figure S3.** Synonymous and non-synonymous substitutional rates of four *Macquartia* species for each PCG. Ks, synonymous substitutional rate; Ka, non-synonymous substitutional rate.
